# Secretory autophagy mediates lysosomal and autophagic degradation for α-synuclein proteostasis

**DOI:** 10.1016/j.jbc.2025.110474

**Published:** 2025-07-17

**Authors:** Taiki Sawai, Yoshitsugu Nakamura, Shigeki Arawaka

**Affiliations:** Division of Neurology, Department of Internal Medicine IV, Osaka Medical and Pharmaceutical University Faculty of Medicine, Osaka, Japan

**Keywords:** autophagy, lysosome, Parkinson disease, proteostasis, protein secretion, synuclein

## Abstract

Autophagy has two distinct pathways, degradation and secretion. Autophagic degradation plays a pivotal role in proteostasis. However, the role of autophagic secretion in proteostasis maintenance is not fully understood. Here, we investigate how the blockade of autophagic secretion impairs proteostasis in SH-SY5Y cells. siRNA-mediated knockdown (KD) of a modulator for autophagosome formation, *ATG5*, *BECN1*, or *FIP200* inhibited autophagic flux and secretion, causing accumulation of Triton X-100-insoluble α-synuclein (α-syn), which is an aggregate-prone protein responsible for neuronal loss in Parkinson’s disease. The blockade of autophagic secretion by KD of t-SNARE *SNAP23* or *STX4* increased autophagic flux for p62 degradation, but these KDs induced enlargement and membrane damage of lysosomes as well as lysosomal dysfunction. *SNAP23* or *STX4* KD caused accumulation of Triton X-100-insoluble α-syn against induction of lysophagy. *GBA* KD showed lysosomal damage with the increase in autophagic secretion. *RAB8A*, a small GTPase regulator of polarized sorting to the plasma membrane, KD blocked autophagic secretion and produced lysosomal damage. *SNAP23*, *STX4*, or *RAB8A* KD further accelerated accumulation of Triton X-100-insoluble α-syn caused by a lysosomal protease inhibitor cocktail. Collectively, these findings suggest that *SNAP23*, *STX4*, or *RAB8A* KD blocks autophagic secretion and upregulates autophagic flux as a compensatory response to help maintain degradation. However, these KDs impair α-syn proteostasis because of lysosomal damage that they induce, counteracting compensatory effects of autophagic degradation, including lysophagy. Autophagic secretion and degradation may collaboratively form the clearance pathway required for maintaining lysosomal function by reducing the burden of aggregate-prone protein cargo.

Autophagy is an evolutionarily conserved pathway for degrading various cytoplasmic materials (the cargo). Autophagic degradation plays a pivotal role in cellular homeostasis against stress and diseases by supplying cellular energy and nutrients during starvation, by maintaining the quality and quantity of intracellular organelles, by eliminating pathogens during infection, and by controlling proteostasis of aggregate-prone proteins to prevent the formation of toxic aggregates (1). The degradative processes are classified into three different types: macroautophagy, microautophagy, and chaperon-mediated autophagy (2). Macroautophagy (hereafter referred to as autophagy) includes a complex series of events that result in the formation of a double-membrane autophagosome and involves the participation of numerous autophagy-related (ATG) proteins (2). During the degradative processes, the cargo is engulfed by autophagosomes in a selective or nonselective bulk manner and is then digested by lysosomal proteases inside the autophagosome–lysosome–fused vesicles, called autolysosomes (1).

There is accumulating evidence that autophagy has an alternative pathway for secretion that involves the extracellular release of the inflammatory cytokine interleukin (IL)-1β and immune mediators (3, 4). The secretory cargos share common features, including the lack of a signal sequence for entering the conventional ER-Golgi route, and they are dependent on ATG proteins for secretion (5). In response to lysosomal damage, IL-1β is trapped by the specialized secretory autophagy cargo receptor TRIM16 on lysosomes and is recruited to the microtubule-associated protein 1 light chain 3 (MAP1LC3/LC3, hereafter referred to as LC3)-II-positive sequestration membrane *via* binding of TRIM16 with the v-SNARE/R-SNARE SEC22B (4). Then, SEC22B is likely transferred to the autophagosome membrane facing the cytosol, where it carries out fusion at the plasma membrane in conjunction with the t-SNARE/Qbc-SNARE SNAP23 or SNAP29 and one of the plasma membrane t-SNAREs/Qa-SNAREs syntaxin (STX) 3 or STX4, eventually secreting IL-1β into the extracellular space. This secretory process is independent of STX17, which promotes autophagosome–lysosome fusion. These observations show that attachment of SEC22B on autophagosomes during lysosomal damage diverts the cargo from the degradative pathway toward the secretory pathway. A previous study has demonstrated that treatment with the lysosomotropic amine chloroquine promotes autophagic secretion, suggesting that the secretory pathway is facilitated as a compensatory response to the blockade of the lysosomal protease supply (6). These findings suggest that autophagy maintains proteostasis by interconnecting the degradative and secretory pathways. However, the role of autophagic secretion in the maintenance of proteostasis remains unclear.

In this study, we hypothesized that autophagy maintained proteostasis by activating the degradative pathway upon the blockade of autophagic secretion. α-Synuclein (α-syn) is one of the pathological aggregate-prone proteins. The formation of α-syn aggregates in cells and the cell-to-cell spread of α-syn aggregates in the brain cause neurodegeneration in Parkinson’s disease (PD) (7). Clarifying the mechanisms that regulate and disrupt α-syn proteostasis is key to understanding the pathogenesis of PD. α-Syn is degraded by chaperon-mediated autophagy and macroautophagy and secreted *via* the autophagic pathway under basal and neuronal activity–induced conditions in neurons (8, 9, 10). Additionally, the intracellular accumulation of Triton X-100-insoluble α-syn is caused by lysosomal damage (11). In this study, we investigated how the blockade of autophagic secretion affected autophagic and lysosomal degradation and proteostatic maintenance by analyzing secretion of α-syn and the accumulation of Triton X-100-insoluble α-syn.

## Results

### Effects of autophagy induction and autophagosome formation on α-syn proteostasis

**Figure 1:**
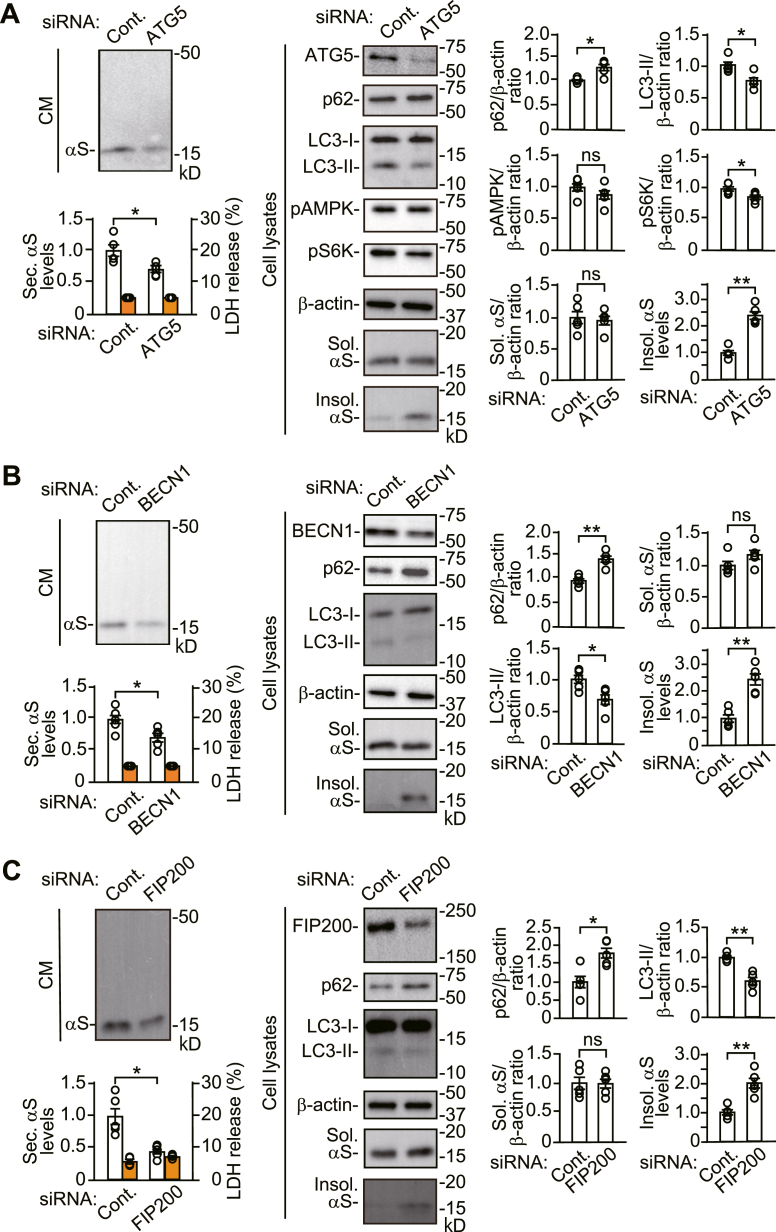
**Effects of *ATG5*, *BECN1, or FIP200* siRNA knockdown on autophagic functions in SH-SY5Y cells stably expressing WT α-syn (wt-αS/SH)**. *A*–*C*, conditioned media (CM) and cell lysates from control, *ATG5* siRNA knockdown (KD) (*A*), *BECN1* siRNA KD (*B*), or *FIP200* siRNA KD (*C*) cells were blotted to detect indicated proteins (n = 5 per group). Lactate dehydrogenase (LDH) release was measured on each condition (n = 4 per group). Graphs show quantitative comparisons of the relative levels and ratios of target proteins. Percentages of LDH release to positive controls are shown as *orange columns*. Data represent mean ± SD. N = 5 or four biological replicates per group (done independently) are plotted for each experiment. Data were analyzed by unpaired *t* test. ∗*p* < 0.05, ∗∗*p* < 0.01. CM, conditioned media; Cont., control; hmCB, heavy-chain mature cathepsin B; Insol. αS, insoluble α-synuclein; ns, not significant; pAMPK, phosphorylated adenosine monophosphate-activated protein kinase; pS6K, phosphorylated ribosomal protein S6 kinase B1; smCB, single-chain mature cathepsin B; Sec. αS, secreted α-synuclein; Sol. αS, soluble α-synuclein.

**Figure 2:**
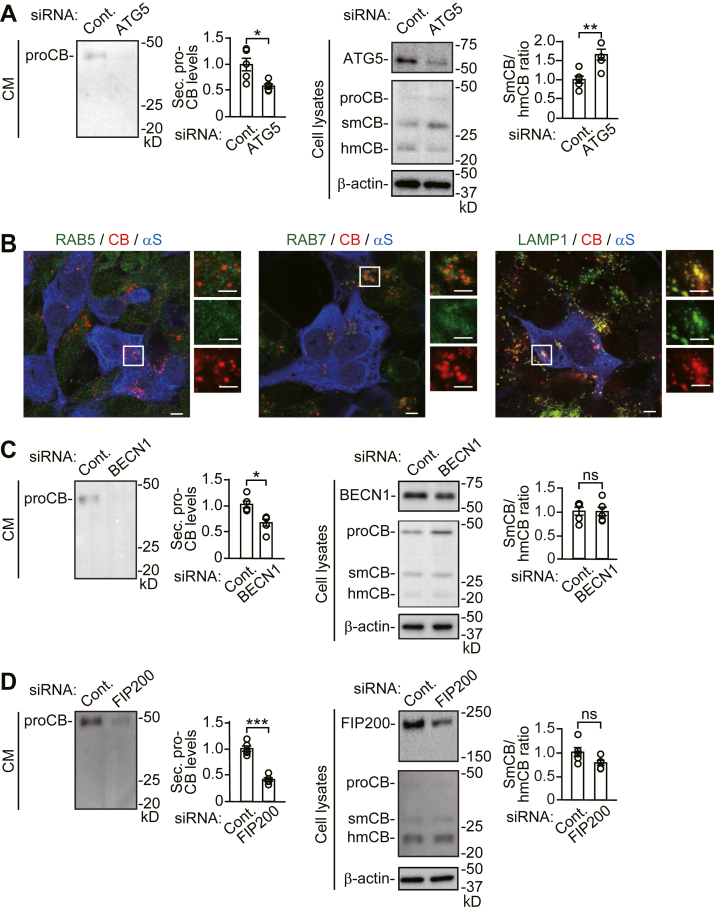
**Effects of *ATG5*, *BECN1, or FIP200* siRNA knockdown on lysosomal functions in wt-αS/SH cells**. *A, C*, and *D*, CM and cell lysates from control, *ATG5* KD (*A*), *BECN1* KD (*C*), or *FIP200* KD (*D*) cells were blotted to detect indicated proteins (n = 5 per group). LDH release was measured on each condition (n = 4 per group). Graphs show quantitative comparisons of the relative levels and ratios of target proteins. *B*, representative fluorescent micrographs from *ATG5* KD cells immunostained for cathepsin B (CB, *red*) and α-synuclein (αS, *blue*) plus RAB5 (*left panels*, *green*), RAB7 (*middle panels*, *green*), or LAMP1 (*right panels*, *green*). Right panels of micrographs show high-magnification images of *white squared areas*. Note that the *top* of *rightpanels* are merged images deleted α-syn staining. Scale bar: 3 μm. Data represent mean ± SD. N = 5 or four biological replicates per group (done independently) are plotted for each experiment. Data were analyzed by unpaired *t* test. ∗*p* < 0.05, ∗∗*p* < 0.01, and ∗∗∗*p* < 0.001. αS, α-synuclein; CB, cathepsin B; CM, conditioned media; Cont., control; hmCB, heavy-chain mature cathepsin B; Insol. αS, insoluble α-synuclein; ns, not significant; proCB, pro-cathepsin B; pS6K, phosphorylated ribosomal protein S6 kinase B1; smCB, single-chain mature cathepsin B; Sec. αS, secreted α-synuclein; Sol. αS, soluble α-synuclein.

To assess a role of autophagic secretion in the maintenance of proteostasis, we analyzed how different autophagy modulator genes affected α-syn secretion and the accumulation of 1% Triton X-100-insoluble α-syn in SH-SY5Y cells. In line with the role of ATG5 that regulates autophagic membrane elongation, siRNA-mediated knockdown (KD) of *ATG5* reduced LC3-II generation and increased intracellular sequestosome 1 (SQSTM1/p62, hereafter referred to as p62) levels, compared with control siRNA, indicating inhibition of basal autophagic flux (Figs. 1*A* and S1) (12). *ATG5* KD reduced α-syn secretion and caused the intracellular accumulation of Triton X-100-insoluble α-syn. siRNA-mediated KD of *BECN1*, which regulates autophagic membrane elongation *via* formation of the class III phosphatidylinositol 3 kinase complex, also reduced basal autophagic flux and α- syn secretion with an intracellular increase in Triton X-100-insoluble α-syn (1) (Figs. 1*B* and S1). These findings were similarly seen in siRNA-mediated KD of adhesion kinase family-interacting protein of 200 kDa (*FIP200*) that regulates autophagy induction *via* formation of the unc-51-like kinase 1/Atg1 complex (13) (Figs. 1*C* and S1). A previous study has shown that cathepsin B (CTSB) undergoes processing for maturation *via* the endosome-lysosome pathway (14). An immature form of CTSB, pro-CTSB, is localized to Golgi apparatus and endosomes and becomes a mature hydrolase inside lysosomes by processing from a single-chain form to a heavy-chain form (14, 15). In control siRNA cells, pro-CTSB was only found as a secretory form of the protein (Fig. 2*A*). Western blots of cell lysates showed that pro-CTSB levels were low, compared with mature single- and heavy-chain CTSB. In *ATG5* siRNA KD cells, immunofluorescent analysis showed that most CTSB signals were colocalized with lysosomal-associated membrane protein 1 (LAMP1, lysosomal marker)-positive structures, and a small number of CTSB signals were colocalized with RAB7-positive (late endosome marker), but not RAB5-positive (early endosome marker), structures (16) (Figs. 2*B* and S2). These findings show that pro-CTSB is secreted *via* an endosomal route or an amphisomal/autophagosomal route and that lysosomal secretion does not occur at detectable levels under basal conditions in SH-SY5Y cells. *ATG5* KD blocked pro-CTSB secretion and CTSB maturation from the single-chain form to the heavy-chain form (Fig. 2*A*). *BECN1* KD and *FIP200* KD also blocked pro-CTSB secretion, but they did not interfere with CTSB maturation from the single-chain form to the heavy-chain form (Fig. 2, *C* and *D*). Collectively, these data show that autophagic processes mediate α-syn and pro-CTSB secretion *via* the amphisomal/autophagosomal route. Additionally, they suggest that inhibition of autophagy induction and autophagosome formation causes the accumulation of Triton X-100-insoluble α-syn, while *ATG5* KD possibly affects it *via* additional function to lysosomes because ATG5 mediates lysosome membrane turnover and lysosome repair (17, 18).

**Figure 3:**
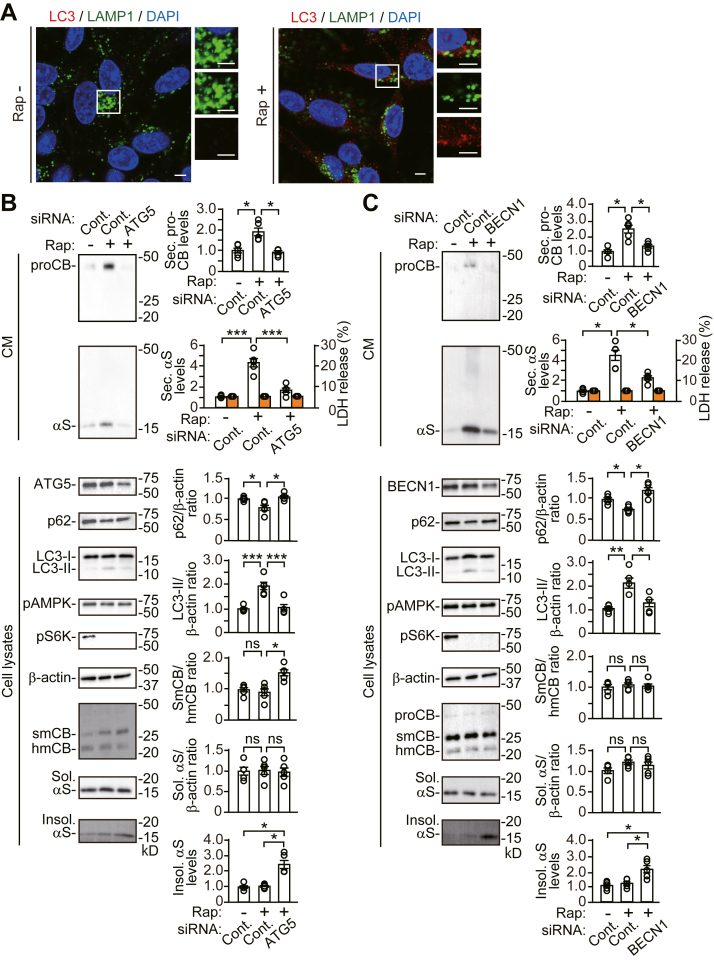
**Effects of *ATG5* or *BECN1* siRNA knockdown on the autophagy-inducible action of rapamycin in wt-αS/SH cells**. Cells were treated with vehicle or 5 μM rapamycin for 24 h. *A*, representative fluorescent micrographs from control cells treated with vehicle (*left images*) or rapamycin (*right images*) immunostained for LC3 (*red*) and LAMP1 (*green*), and nuclei (DAPI, *blue*). Left micrographs show merged images. Right panels of micrographs show images of *white squared areas*. Scale bar: 3 μm. *B* and *C*, CM and cell lysates of control, *ATG5* KD (*B*), or *BECN1* KD (*C*) cells were blotted to detect indicated proteins (n = 5 per group). LDH release was measured on each condition (n = 4 per group). Graphs show quantitative comparisons of the relative levels and ratios of target proteins. Percentages of LDH release to positive controls are shown as *orange columns*. Data represent mean ± SD. N = 5 or four biological replicates per group (done independently) are plotted for each experiment. Data were analyzed by Welch ANOVA with Games-Howell *post hoc* tests in secreted pro-cathepsin B and insoluble α-synuclein (α-syn) levels of (*B*) and in secreted α-syn levels and LC3-II/β-actin ratios of (*C*). Other data were analyzed by one-way ANOVA with Bonferroni *post hoc* tests. ∗*p* < 0.05, ∗∗*p* < 0.01, ∗∗∗*p* < 0.001. CM, conditioned media; Cont., control; hmCB, heavy-chain mature cathepsin B; Insol. αS, insoluble α-synuclein; ns, not significant; proCB, pro-cathepsin B; pAMPK, phosphorylated adenosine monophosphate-activated protein kinase; pS6K, phosphorylated ribosomal protein S6 kinase B1; rap, rapamycin; smCB, single-chain mature cathepsin B; Sec. αS, secreted α-synuclein; Sol. αS, soluble α-synuclein.

To further address the relationship between autophagosome formation and lysosomal function, we analyzed the effects of *ATG5* KD on lysosomal function under rapamycin-induced autophagy. Treatment with rapamycin increased LC3-positive puncta and their colocalization with LAMP1-positive structures, compared with nontreated cells, showing that rapamycin induced autophagosome and autolysosome formation (Figs. 3*A* and S3). In control siRNA cells, treatment with rapamycin blocked phosphorylation of ribosomal protein S6 kinase B1 (S6K) without altering phosphorylation of adenosine monophosphate-activated protein kinase, and it increased intracellular LC3-II generation with a reduction in intracellular p62 levels, indicating induction of autophagic flux for degradation *via* inhibition of the mechanistic target of rapamycin complex 1 (mTORC1) signaling pathway (Fig. 3*B*). Rapamycin promoted pro-CTSB secretion without altering CTSB maturation from the single-chain form to the heavy-chain form. Rapamycin also promoted α-syn secretion without altering the intracellular levels of soluble or Triton X-100-insoluble α-syn. These findings show that while rapamycin promotes autophagic secretion, the maturation of CTSB, and the intracellular levels of soluble, and Triton X-100-insoluble α-syn are unaffected by the increase in autophagic flux. However, *ATG5* KD interfered with the ability of rapamycin to stimulate autophagic flux for degradation, although rapamycin inhibited the phosphorylation of S6K (Fig. 3*B*). Moreover, *ATG5* KD interfered with rapamycin-stimulated secretion of α-syn and pro-CTSB secretion. *ATG5* KD blocked CTSB maturation and caused the intracellular accumulation of Triton X-100-insoluble α-syn. *BECN1* KD also interfered with the ability of rapamycin to stimulate autophagic flux for degradation and secretion of α-syn and pro-CTSB. *BECN1* KD caused the intracellular accumulation of Triton X-100-insoluble α-syn, but it did not affect CTSB maturation from the single-chain form to the heavy-chain form (Fig. 3*C*). These findings support that inhibition of autophagosome formation sufficiently causes the accumulation of Triton X-100-insoluble α-syn, while *ATG5* KD affects α-syn proteostasis *via* the suppression of additional function to lysosomes (17, 18).

### SNAP23 or STX4 KD alters autophagic secretion and degradation with the enlargement and membrane damage of lysosomes

**Figure 4:**
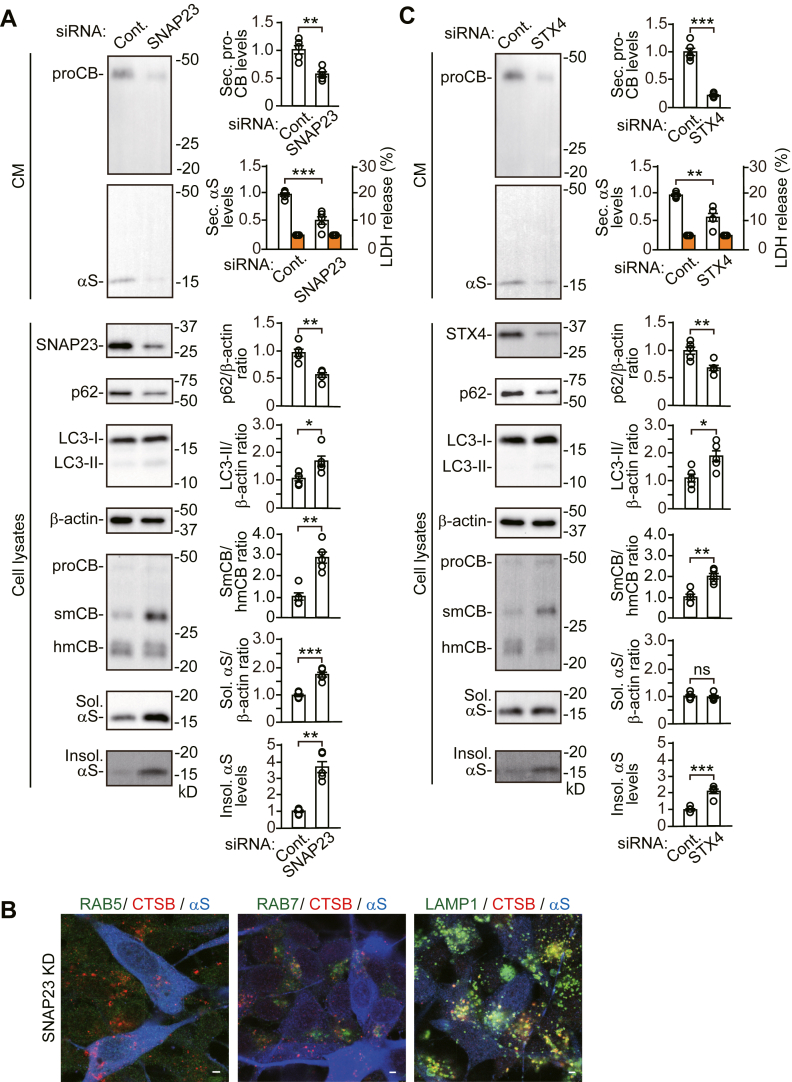
**Effects of *SNAP23* or *STX4* siRNA knockdown on autophagic and lysosomal functions in wt-αS/SH cells**. *A* and *C*, CM and cell lysates from control, *SNAP23* KD (*A*), or *STX4* KD (*C*) cells were blotted to detect indicated proteins (n = 5 per group). LDH release was measured on each condition (n = 4 per group). Graphs show quantitative comparisons of the relative levels and ratios of target proteins. Percentages of LDH release to positive controls are shown as *orange columns*. *B*, representative fluorescent micrographs from *SNAP23* KD cells immunostained for cathepsin B (CTSB, *red*) and α-syn (αS, *blue*) plus RAB5 (*left panel*, *green*), RAB7 (*middle panel*, *green*), or LAMP1 (*right panel*, *green*). Scale bar: 3 μm. Data represent mean ± SD. N = 5 or four biological replicates per group (done independently) are plotted for each experiment. Data were analyzed by unpaired *t* test. ∗*p* < 0.05, ∗∗*p* < 0.01, ∗∗∗*p* < 0.001. αS, α-synuclein; CM, conditioned media; Cont., control; hmCB, heavy-chain mature cathepsin B; Insol. αS, insoluble α-synuclein; ns, not significant; proCB, pro-cathepsin B; smCB, single-chain mature cathepsin B; Sec. αS, secreted α-synuclein; Sol. αS, soluble α-synuclein.

To assess how autophagic secretion affects autophagic and lysosomal degradation, we transfected cells with siRNAs against *SNAP23* or *STX4*, a t-SNARE that modulates fusion of membrane vesicles with the plasma membrane (4). *SNAP23* KD reduced pro-CTSB and α-syn secretion (Fig. 4*A*). The reduced α-syn secretion included soluble and Triton X-100-insoluble forms (Fig. S4). *SNAP23* KD increased LC3-II generation, while reducing intracellular p62 levels, compared with control siRNA (Fig. 4*A*). Additionally, *SNAP23* KD inhibited CTSB maturation from the single-chain form to the heavy-chain form. Immunofluorescent analysis showed that *SNAP23* KD did not affect the localization of CTSB (Fig. 4*B*). CTSB was mainly colocalized to LAMP1-positive structures, and there was no colocalization of CTSB with RAB5-positive structures. These findings suggest that *SNAP23* KD blocked autophagic secretion with promoting autophagic flux for degradation and inducing lysosomal dysfunction. In line with this, *SNAP23* KD caused impairment of α-syn proteostasis, as shown by the intracellular accumulation of soluble and Triton X-100-insoluble α-syn (Fig. 4*A*). To assess the effects of *SNAP23* KD on autophagic and lysosomal functions, we treated *SNAP23* KD cells with bafilomycin A1, a chemical inhibitor for lysosomal acidification and autophagosome–lysosome fusion (19). Treatment with bafilomycin A1 inhibited *SNAP23* KD-induced promotion of autophagic degradation for p62, and it further accelerated *SNAP23* KD-induced generation of LC3-II (Fig. S5). Bafilomycin A1 did not alter the *SNAP23* KD-induced blockade of CTSB maturation from the single-chain form to the heavy-chain form. These findings suggest that *SNAP23* KD promotes autophagic flux for degradation and causes lysosomal dysfunction in response to the blockade of autophagic secretion. *STX4* KD also blocked pro-CTSB and α-syn secretion, and it promoted autophagic flux for degradation (Fig. 4*C*). *STX4* KD increased the intracellular accumulation of Triton X-100-insoluble α-syn, although to a lesser extent than *SNAP23* KD, without altering the intracellular levels of soluble α-syn.

**Figure 5:**
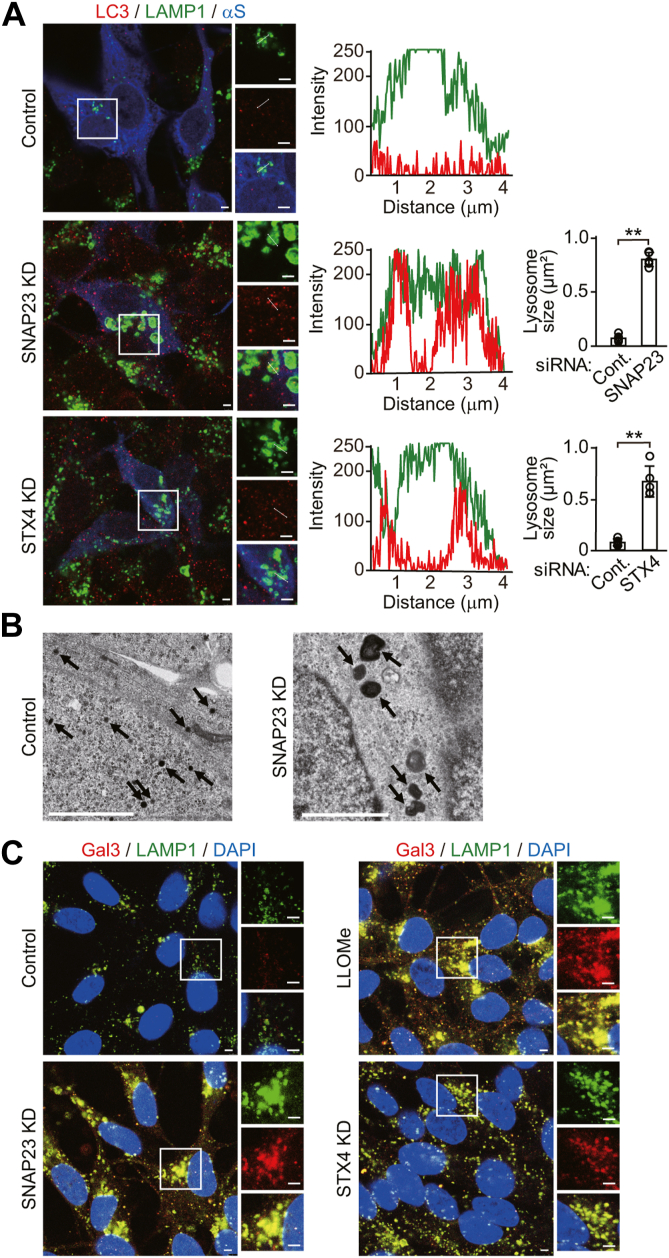
**Effects of *SNAP23* or *STX4* siRNA knockdown on lysosome morphology and lysophagy in wt-αS/SH cells**. *A*, representative fluorescent micrographs from control, *SNAP23* or *STX4* KD cells immunostained for LC3 (*red*), LAMP1 (*green*), and α-syn (αS, *blue*). The line graphs show *c*olocalization line tracing analysis from left images. The bar graphs show comparison of the lysosome size between control and siRNA KD cells. The values were estimated by measuring the size of LAMP1-positive structures in five cells selected arbitrarily. Data represent mean ± SD and were analyzed by unpaired *t* test. ∗∗*p* < 0.01. *B*, transmission electron microscopic images from control and *SNAP23* KD cells are shown. *Arrows* indicate lysosomes. Scale bar: 2 μm. *C*, representative fluorescent micrographs from control or *SNAP23* KD cells immunostained for galectin 3 (Gal3, *red*), LAMP1 (*green*), and nuclei (DAPI, *blue*). *A* and *C*, right panels of micrographs show images of *white squared areas*. Scale bar: 3 μm. αS, α-synuclein; Gal3, galectin 3; LLOMe, L-leucyl-L-leucine methyl ester.

We then analyzed the morphological changes in lysosomes in *SNAP23* or *STX4* KD cells by immunofluorescence. In *SNAP23* KD cells, many LAMP1-potive structures significantly enlarged, compared with control siRNA cells (Fig. 5*A*). Transmission electron microscopic analysis showed that *SNAP23* KD caused the enlargement of lysosomes, compared with control siRNA cells (Fig. 5*B*). This enlargement of LAMP1-positive structures was also seen in *STX4* KD cells but to a lesser extent. Additionally, the enlarged LAMP1-positive structures contained LC3-positive puncta in *SNAP23* or *STX4* KD cells (Fig. 5*A*). These findings show that the *SNAP23* or *STX4* KD induces enlargement of lysosomes and lysophagy for the removal of damaged lysosomes. To test how lysophagy is triggered by these KDs, we examined the recruitment of galectin 3 in membrane-damaged lysosomes (20, 21). In nontreated cells, galectin 3-positive signals were faint, and there was no colocalization with LAMP1-positive structures (Fig. 5*C*). In contrast, treatment with the lysosomotropic reagent L-leucyl-L-leucine methyl ester caused colocalization of enlarged LAMP1-positive structures with galectin 3-positive puncta (20). *ATG5* KD also induced colocalization of LAMP1-positive structures with galectin 3 (Fig. S6). In *SNAP23* or *STX4* KD cells, enlarged LAMP1-positive structures colocalized with galectin 3-positive puncta. In *SNAP23* KD cells, LAMP1-positive structures colocalized with LC3 and galectin 3-positive puncta (Fig. S7). Collectively, these findings show that the *SNAP23* or *STX4* KD causes membrane damage to lysosomes, possibly triggering lysophagy.

**Figure 6:**
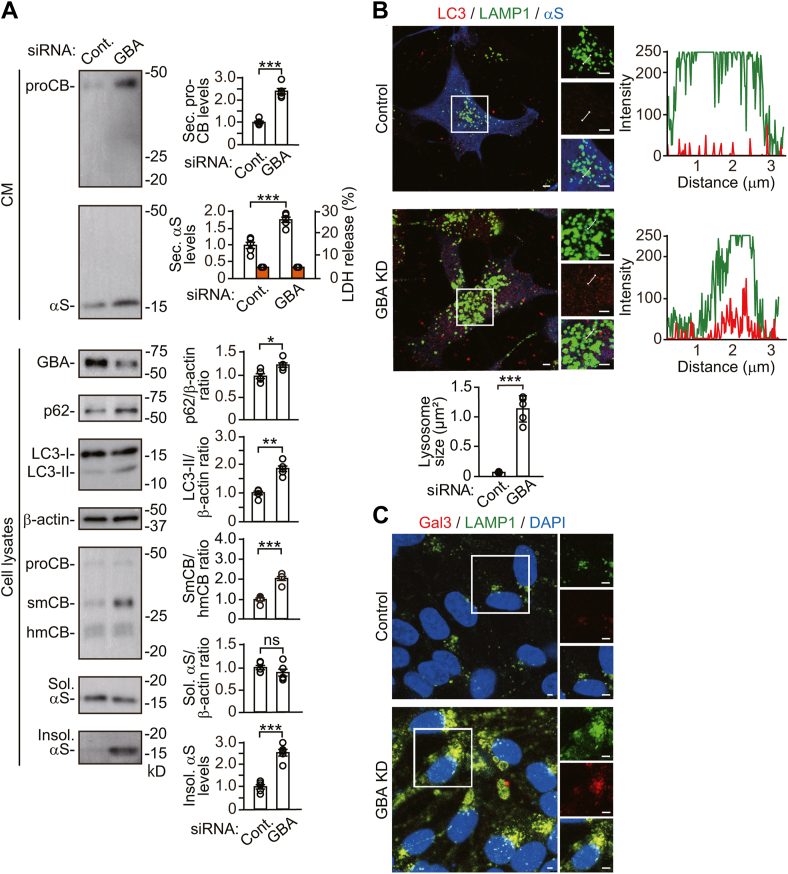
**Effects of *GBA* siRNA knockdown on autophagic and lysosomal functions in wt-αS/SH cells**. *A*, CM and cell lysates from control or *GBA* KD cells were blotted to detect indicated proteins (n = 5). LDH release was measured on each condition (n = 4). Graphs show quantitative comparisons of the relative levels and ratios of target proteins. Percentages of LDH release to positive controls are shown as *orange columns*. *B*, representative fluorescent micrographs from control or *GBA* KD cells immunostained for LC3 (*red*), LAMP1 (*green*), and α-syn (αS, *blue*). The line graphs show *c*olocalization line tracing analysis from left images. The bar graph shows comparison of the lysosome size between control and *GBA* KD cells. The values were estimated by measuring the size of LAMP1-positive structures in five cells selected arbitrarily. *C*, representative fluorescent micrographs from control or *GBA* KD cells immunostained for galectin 3 (Gal3, *red*), LAMP1 (*green*), and nuclei (DAPI, *blue*). *B* and *C*, right panels of micrographs show images of *white squared areas*. Scale bar: 3 μm. Data represent mean ± SD. N = 5 or four biological replicates per group (done independently) are plotted for each experiment. Data were analyzed by unpaired *t* test. ∗*p* < 0.05, ∗∗*p* < 0.01, ∗∗∗*p* < 0.001. αS, α-synuclein; CM, conditioned media; Cont., control; Gal3, galectin 3; hmCB, heavy-chain mature cathepsin B; Insol αS, insoluble α-synuclein; proCB, pro-cathepsin B; smCB, single-chain mature cathepsin B; Sec. αS, secreted α-synuclein; Sol. αS, soluble α-synuclein.

To test whether *SNAP23* or *STX4* KD causes lysosome damage independently of the blockade of autophagic secretion, we examined cells transfected with siRNAs against *GBA*, which encodes a lysosomal enzyme, β-glucocerebrosidase, and whose variants increase susceptibility to PD (22). *GBA* KD blocked CTSB maturation from the single-chain form to the heavy-chain form and caused the accumulation of Triton X-100-insoluble α-syn (Fig. 6*A*). Additionally, *GBA* KD increased intracellular p62 levels, while promoting α-syn secretion. We then analyzed the morphological changes in lysosomes caused by *GBA* KD. In *GBA* KD cells, many LAMP1-positive structures were enlarged, compared with control siRNA cells, and the enlarged LAMP1-positive structures contained LC3-positive puncta (Fig. 6*B*). Enlarged LAMP1-positive structures colocalized with galectin 3-positive puncta in *GBA* KD cells, compared with control siRNA cells (Fig. 6*C*). These findings demonstrate that lysosomal dysfunction and damage emerge as impairment of autophagic degradation and promotion of autophagic secretion. They suggest that promotion of autophagic degradation seen in *SNAP23* or *STX4* KD is unexplainable only by lysosome damage, and it represents a compensatory response to the blockade of autophagic secretion.

### SNAP23 or STX4 KD accelerates impairment of α-syn proteostasis caused by lysosomal protease inhibition

**Figure 7:**
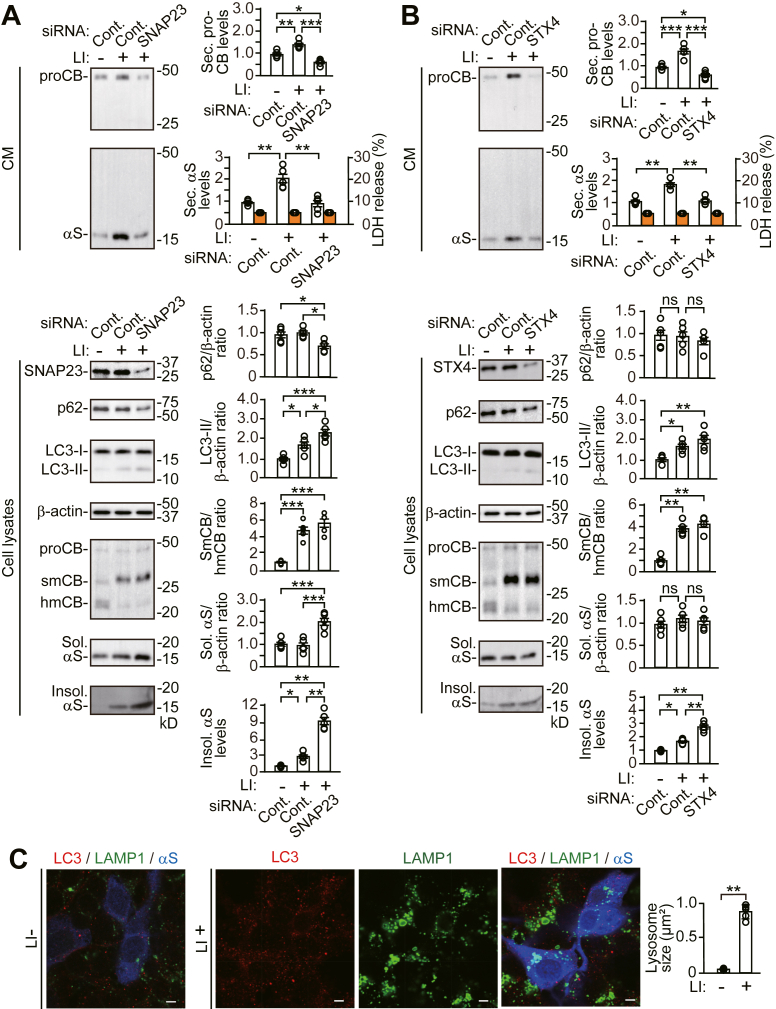
**Effects of *SNAP23* or *STX4* siRNA knockdown on the action of the lysosomal protease inhibitor cocktail in wt-αS/SH cells**. Cells were treated with vehicle or the lysosomal protease inhibitor cocktail (E64-days, Pepstatin A, and Leupeptin at 5 μg/dl each) for 24 h. *A* and *B*, CM and cell lysates from *SNAP23* KD (*A*) or *STX4* KD (*B*) cells were blotted to detect indicated proteins (n = 5 per group). LDH release was measured on each condition (n = 4 per group). Graphs show quantitative comparisons of the relative levels and ratios of target proteins. Percentages of LDH release to positive controls are shown as *orange columns*. Data represent mean ± SD. N = 5 or four biological replicates per group (done independently) are plotted for each experiment. Data were analyzed by Welch ANOVA with Games–Howell *post hoc* tests in secreted α-syn and insoluble α-syn levels of (*A*) and in insoluble α-syn levels of (*B*). Other data were analyzed by one-way ANOVA with Bonferroni *post hoc* tests. *C*, representative fluorescent micrographs from wt-αS/SH cells treated with vehicle or the protease inhibitor cocktail for 24 h immunostained for LC3 (*red*) and LAMP1 (*green*), and α-syn (αS, *blue*). Scale bar: 3 μm. The bar graph shows comparison of the lysosome size between cells with and without the lysosome protease inhibitor cocktail treatment. The values were estimated by measuring the size of LAMP1-positive structures in five cells selected arbitrarily. ∗*p* < 0.05, ∗∗*p* < 0.01, ∗∗∗*p* < 0.001. αS, α-synuclein; CM, conditioned media; Cont., control; hmCB, heavy-chain mature cathepsin B; Insol. αS, insoluble α-synuclein; LI, lysosomal protease inhibitor; ns, not significant; proCB, pro-cathepsin B; smCB, single-chain mature cathepsin B; Sec. αS, secreted α-synuclein; Sol. αS, soluble α-synuclein.

To assess how autophagic secretion affects α-syn proteostasis in relation to autophagic and lysosomal degradation, we investigated it under inhibition of lysosomal proteases. Treatment with a lysosomal protease inhibitor cocktail increased LC3-II generation, although intracellular p62 levels were unaffected (Fig. 7*A*). The inhibitor treatment promoted secretion of α-syn and pro-CTSB and the intracellular accumulation of Triton X-100-insoluble α-syn, without altering intracellular levels of soluble α-syn. *SNAP23* KD interfered with the lysosomal protease inhibitor cocktail-induced increase in α-syn and pro-CTSB secretion. *SNAP23* KD increased LC3-II generation with reduction in intracellular p62 levels, showing promotion of autophagic flux for degradation against inhibition of lysosomal proteases. The intracellular accumulation of Triton X-100-insoluble α-syn in *SNAP23* KD cells was more abundant than that in control siRNA cells under inhibition of lysosomal proteases. Intracellular levels of soluble α-syn were also increased by *SNAP23* KD. Similarly, *STX4* KD reduced the increase in α-syn and pro-CTSB secretion by the protease inhibitor cocktail (Fig. 7*B*). The intracellular accumulation of Triton X-100-insoluble α-syn was increased in *STX4* KD cells, compared with control siRNA cells, under inhibition of lysosomal proteases. Immunofluorescence analysis showed that the lysosomal protease inhibitor cocktail induced LC3-positive puncta and enlargement of LAMP1-positive structures, and a subset of the enlarged LAMP1-positive structures contained LC3-positive puncta (Fig. 7*C*). These results show that autophagic secretion helps maintain α-syn proteostasis against lysosomal protease inhibition.

### RAB8A KD blocks autophagic secretion with inducing lysosomal damage, lysophagy, and impairment of α-syn proteostasis

**Figure 8:**
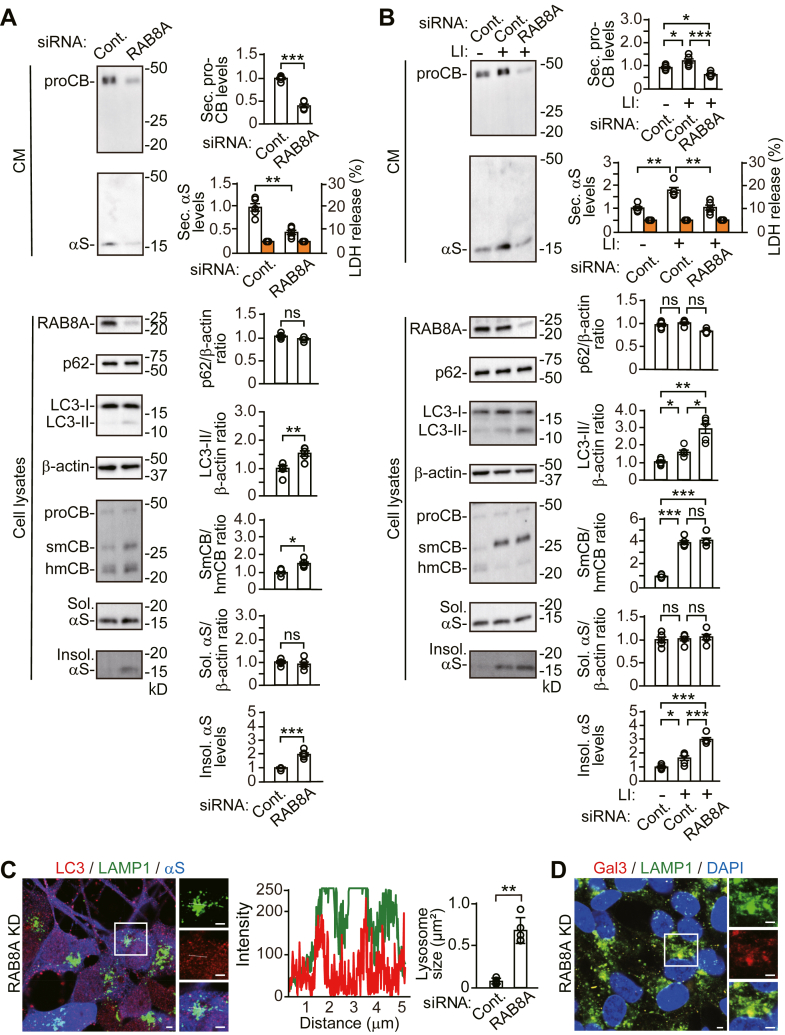
**Effects of *RAB8A* siRNA knockdown on autophagic and lysosomal functions in wt-αS/SH cells**. *A* and *B*, CM and cell lysates from control or *RAB8A* KD cells were blotted to detect indicated proteins (n = 5 per group). LDH release was measured on each condition (n = 4 per group). Graphs show quantitative comparisons of the relative levels and ratios of target proteins. Percentages of LDH release to positive controls are shown as *orange columns*. *B*, control or *RAB8A* KD cells were treated with vehicle or the lysosomal protease inhibitor cocktail for 24 h. Data represent mean ± SD. N = 5 or four biological replicates per group (done independently) are plotted for each experiment. Data were analyzed by unpaired *t* test (*A*) and Welch ANOVA with Games–Howell *post hoc* tests in LC3-II/β-actin ratios of (*B*). Other data of (*B*) were analyzed by one-way ANOVA with Bonferroni *post hoc* tests. *C*, representative fluorescent micrographs from *RAB8A* KD cells immunostained for LC3 (*red*), LAMP1 (*green*), and α-syn (αS, *blue*) The line graph shows *c*olocalization line tracing analysis from left images. The bar graph shows comparison of the lysosome size between control and *RAB8A* KD cells. The values were estimated by measuring the size of LAMP1-positive structures in five cells selected arbitrarily. *D*, fluorescent micrographs from *RAB8A* KD cells immunostained for galectin 3 (Gal3, *red*), LAMP1 (*green*), and nuclei (DAPI, *blue*). *C* and *D*, right panels of micrographs show images of *white squared areas*. Scale bar: 3 μm. ∗*p* < 0.05, ∗∗*p* < 0.01, ∗∗∗*p* < 0.001. αS, α-synuclein; CM, conditioned media; Cont., control; Gal3, galectin 3; hmCB, heavy-chain mature cathepsin B; Insol. αS, insoluble α-synuclein; LI, lysosomal protease inhibitor; ns, not significant; proCB, pro-cathepsin B; smCB, single-chain mature cathepsin B; Sec. αS, secreted α-synuclein; Sol. αS, soluble α-synuclein.

To explore the mechanism by which autophagic secretion affects autophagic and lysosomal degradation, we investigated cells treated with siRNAs against *RAB8A*, a small GTPase protein that regulates polarized sorting to the plasma membrane and mediates fusion of autophagosomes with the plasma membrane during autophagic secretion (23, 24). *RAB8A* KD reduced pro-CTSB and α-syn secretion, compared with control siRNA (Fig. 8*A*). *RAB8A* KD slightly increased LC3-II generation, without altering intracellular p62 levels. *RAB8A* KD inhibited CTSB maturation from the single-chain form to the heavy-chain form and caused the intracellular accumulation of Triton X-100-insoluble α-syn, without altering intracellular levels of soluble α-syn. *RAB8A* KD interfered with the ability of the lysosome protease inhibitor cocktail to stimulate α-syn and pro-CTSB secretion (Fig. 8*B*). *RAB8A* KD accelerated the intracellular accumulation of Triton X-100-insoluble α-syn caused by the lysosome protease inhibitor cocktail, while intracellular levels of soluble α-syn were unaffected. Immunofluorescent analysis showed that *RAB8A* KD caused enlargement of LAMP1-positive structures, compared with control siRNA cells (Fig. 8*C*). These enlarged LAMP1-positive structures contained LC3-positive puncta. Additionally, the enlarged LAMP1-positive structures colocalized with galectin 3-positive puncta (Fig. 8*D*). The results suggest that the *RAB8A* KD-induced blockade of autophagic secretion coincides lysosomal damage and lysophagy and impairs α-syn proteostasis. Golgi reassembly stacking protein 55 (GRASP55) promotes autophagic secretion *via* relocalization of GRASP55 from Golgi to autophagosomes and late endosomes in response to mTORC1 inactivation (25, 26). Additionally, GRASP55 mediates fusion between autophagosomes and lysosomes (25, 27). We analyzed the effects of *GRASP55* KD on α-syn proteostasis and lysosomal function. *GRASP55* KD reduced pro-CTSB and α-syn secretion, compared with control siRNA (Fig. S8). *GRASP55* KD did not alter LC3-II generation, but it increased intracellular p62 levels, showing the suppression of autophagic degradation. *GRASP55* KD caused the intracellular accumulation of Triton X-100-insoluble α-syn, without altering intracellular levels of soluble α-syn, but it maintained lysosomal function as shown in intact CTSB maturation from the single-chain form to the heavy-chain form. The results may be explained by a scenario that *GRASP55* KD-induced disconnection between autophagosomes and lysosomes impedes autophagic degradation, while keeping lysosomes intact. They suggest that the blockade of autophagic secretion affects lysosomal function *via* interconnection between autophagosomes and lysosomes.

### The SNAP23 or RAB8A KD-induced blockade of autophagic secretion impairs α-syn proteostasis against the autophagy-inducible action of rapamycin

**Figure 9:**
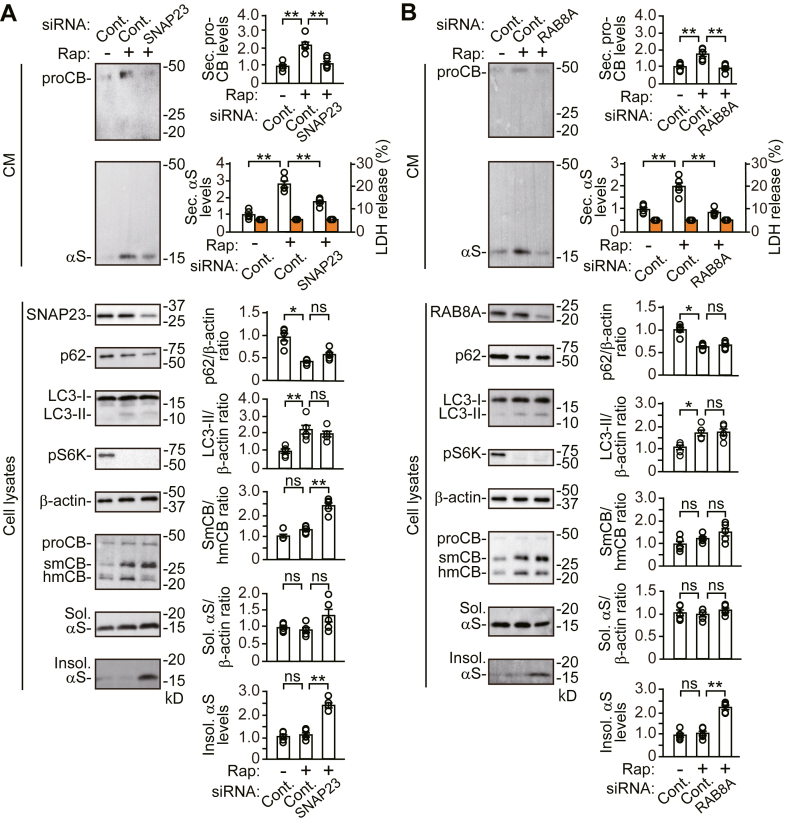
**Effects of *SNAP23* or *RAB8A* siRNA knockdown on the autophagy-inducible action of rapamycin in wt-αS/SH cells**. Cells were treated with vehicle or 5 μM rapamycin for 24 h. *A* and *B*, CM and cell lysates from control, *SNAP23* KD (*A*), or *RAB8A* KD (*B*) cells were blotted to detect indicated proteins (n = 5 per group). LDH release was measured on each condition (n = 4 per group). Graphs show quantitative comparisons of the relative levels and ratios of target proteins. Percentages of LDH release to positive controls are shown as *orange columns*. Data represent mean ± SD. N = 5 or four biological replicates per group (done independently) are plotted for each experiment. Data were analyzed by Welch ANOVA with Games–Howell *post hoc* tests in p62 and soluble α-syn levels of (*A*). Other data were analyzed by one-way ANOVA with Bonferroni *post hoc* tests. ∗*p* < 0.05, ∗∗*p* < 0.01. CM, conditioned media; Cont., control; hmCB, heavy-chain mature cathepsin B; Insol. αS, insoluble α-synuclein; ns, not significant; proCB, pro-cathepsin B; pS6K, phosphorylated ribosomal protein S6 kinase B1; rap, rapamycin; smCB, single-chain mature cathepsin B; Sec. αS, secreted α-synuclein; Sol. αS, soluble α-synuclein.

To assess whether the blockade of autophagic secretion counteracts a compensatory response of autophagic degradation on α-syn proteostasis, we investigated the effects of *SNAP23* or *RAB8A* KD on rapamycin-induced autophagy. As shown in Figure 3, treatment with rapamycin promoted autophagic flux for degradation *via* mTORC1 signaling, as indicated by the increase in intracellular LC3-II levels and the reduction in intracellular p62 levels. Treatment with rapamycin also promoted autophagic secretion of pro-CTSB and α-syn. *SNAP23* KD had no effect on rapamycin-stimulated mTORC1 signaling (Fig. 9*A*). However, *SNAP23* KD interfered with the ability of rapamycin to stimulate α-syn and pro-CTSB secretion. *SNAP23* KD caused the intracellular accumulation of Triton X-100-insoluble α-syn despite the increase in autophagic flux for degradation. These findings were also seen in *RAB8A* KD cells (Fig. 9*B*). *SNAP23* KD inhibited the processing of mature CTSB, although *RAB8A* KD did not in this experiment (Fig. 9, *A* and *B*). These findings show that the *SNAP23* or *RAB8A* KD-induced blockade of autophagic secretion counteracts the increase in autophagic flux for degradation on α-syn proteostasis.

## Discussion

Autophagy plays a central role in maintaining proteostasis *via* the degradative and secretory pathways. Autophagic secretion is upregulated in response to pharmacological inhibition of lysosomal function by the lysosome acidification inhibitor bafilomycin A or the lysosomotropic amine chloroquine (6). Autophagic secretion plays a role in clearance of damaged mitochondria, and the deficiency of mammalian ATG8 lipidation may redirect autophagosome trafficking toward the plasma membrane instead of lysosomes (28). Autophagic secretion is also induced by shRNA-mediated KD of *SNAP29* or *VAMP8*, which is required for autophagosome–lysosome fusion (29). Autophagic secretion acts as a compensatory mechanism against inhibition of autophagic and lysosomal degradation (6). However, it is unclear whether autophagic and lysosomal degradation are upregulated in response to the blockade of autophagic secretion. In this study, we found that the *SNAP23* or *STX4* KD-induced blockade of autophagic secretion promoted autophagic degradation for the autophagy cargo receptor p62 and that *SNAP23* or *STX4* KD caused functional and morphological damage to lysosomes and the accumulation of Triton X-100-insoluble α-syn. These findings show that autophagic degradation is upregulated as a compensatory response to the blockade of autophagic secretion but that lysosomal damage nonetheless occurs and counteracts the effects of the increased autophagic degradation. We propose that autophagy involves interactions between the degradative and secretory pathways and that autophagic secretion is required for lysosomal quality control by reducing cargo overload, including aggregate-prone proteins, to lysosomes. In support of this model, the present data show that the *SNAP23*, *STX4*, or *RAB8A* KD-induced blockade of autophagic secretion accelerated α-syn proteostasis impairment by pharmacological inhibition of lysosomal proteases, showing that the blockade of autophagic secretion *per se* causes cargo overload.

Although it is possible that colocalization of LAMP1-positive structures with galectin 3 represented endosomal sorting complex required for transport–mediated membrane repair in damaged lysosomes, the present immunofluorescence analysis showed that a subset of damaged lysosomes where galectin 3 was recruited directed toward lysophagy (30). A previous study has demonstrated that lysophagy protects against lysosomal damage–induced propagation of α-syn aggregates in cells treated with exogenous preformed α-syn fibrils (21). The protective effects of lysophagy on α-syn proteostasis may be affected by the remaining degradative activity of intact lysosomes and the residual autophagic secretion that removes monomeric and aggregated α-syn to the extracellular space. In addition to lysophagy, membrane-damaged lysosomes are replaced, removed, and repaired by multiple pathways, including autophagic lysosome reformation (31), the transcription factor EB–associated lysosomal biogenesis pathway (32, 33), microautophagy, endosomal sorting complex required for transport-mediated lysosomal repair pathway (30, 34), and the phosphoinositide-initiated membrane tethering and lipid transport pathway (35). It is necessary to further assess how the blockade of autophagic secretion influences these pathways involved in lysosomal quality control.

Here, we found that *SNAP23* or *STX4* KD increased autophagic degradation, as indicated by a reduction in p62 levels, while *RAB8A* KD did not alter p62 levels. T-SNAREs, SNAP23 and STX4, mediate the fusion of various vesicles with the plasma membrane. The vesicles include late endosomes and lysosomes, as well as autophagosomes and amphisomes (36, 37). RAB8 modulates apical exocytosis in epithelial cells, and RAB8A mediates fusion of autophagosomes and amphisomes with the plasma membrane in neurons and inflammatory cells (23, 38, 39, 40, 41). Autophagic degradation of p62 may be induced in a manner dependent on the strength of the blockade of secretory pathways. *SNAP23* or *STX4* KD may block a broader range of exocytotic pathways (*e.g.*, endolysosomal exocytosis) than *RAB8A* KD. Alternatively, *SNAP23* or *STX4* KD may block autophagosome–plasma membrane fusion more potently than *RAB8A* KD. Moreover, we found that *SNAP23* KD reduced p62 levels in cells treated with the lysosomal protease inhibitor cocktail, while single treatment with the inhibitor cocktail did not alter p62 levels. Although it remains unknown how *SNAP23* KD can clear p62 despite lysosomal damage and the pharmacological inhibition of lysosomal proteases, intact lysosomes containing residual active proteases may fuse with autophagosomes for maintaining autophagic degradation of p62 in response to the *SNAP23* KD-induced blockade of autophagic secretion.

The role of autophagic secretion has become increasingly clear. In the modulation of synaptic plasticity, neuronal activity enhances autophagic secretion in concert with SNAP29, SEC22, and RAB8 but diminishes autophagic degradation in *Drosophila* (42). The results indicate that autophagic secretion and degradation are divergent pathways that are regulated by the interplay of SNAREs and RAB. STX17 has been shown to promote autophagic degradation by catalyzing autophagosome–lysosome fusion (29). These results provide further support for the presence of compensatory interactions between autophagic secretion and degradation, and they raise the possibility that autophagic degradation can be modulated by inhibiting or activating SNAREs and RAB (43). Upregulating autophagic degradation may have application in PD therapy *via* enhancing the clearance of α-syn aggregates. Conversely, downregulating autophagic secretion may provide a different therapeutic approach to slow cell-to-cell transmission of α-syn by suppressing secretion of protein seeds. However, it is unclear whether or not cellular response, such as lysosome damage and α-syn proteostasis impairment, to the blockade of autophagic secretion is more beneficial for cell survival than a decrease in cell-to-cell transmission of α-syn. It should be noted that this study provided no data on how the *SNAP23*, *STX4*, or *RAB8A* KD-induced blockade of autophagic secretion affected cell survival, because the accumulation of 1% Triton X-100-inslolube α-syn had no toxicity to cells during our experiments as shown by lactate dehydrogenase (LDH) assays. Degradative function of the autophagy–lysosome pathway is revealed to deteriorate with aging and to associate with the pathogenesis of various neurodegenerative diseases (44, 45). Autophagic secretion may participate in proteostasis to clear aggregate-prone proteins related to neurodegenerative diseases. Further studies are warranted to elucidate the mechanisms regulating autophagic secretion and degradation as well as the processes that maintain proteostasis of aggregate-prone proteins in disease models.

## Experimental procedures

### Materials

Reagents for cell culture experiments and chemical reagents were obtained from Thermo Fisher Scientific and Sigma-Aldrich, respectively, unless otherwise stated.

### Cell culture, siRNA transfection, and LDH assay

We used an SH-SY5Y cell line (origin, #ECACC 94030304) stably expressing WT α-syn (wt-αS/SH) (46). The cells were maintained in Eagle’s minimum essential medium/Ham’s F-12 (Sigma-Aldrich) supplemented with 15% FBS, 2 mM L-glutamine, and 1× nonessential amino acids (Sigma-Aldrich).

For siRNA-mediated KD, approximately 30% confluent cells in 6-well plates were transfected with siRNA oligonucleotides (final concentration at 1 nM, Silencer Select RNAi) using RNAiMAX reagent according to the manufacturer's protocol (47). The sequences of siRNA oligonucleotides used were as follows: *ATG5* (GGAUGCAAUUGAAGCUCAU), *BECN1* (GCAGUUCAAAGAAGAGGUU), *STX4* (CAGCAAUUCGUGGAGCUCA), *SNAP23* (CAGAGAUCGUAUUGAUAUU), *RAB8A* (GAGUCAAAAUCACACCGGA), *FIP200* (GCCUAGAACAACUAACGAA), *GRASP55* (CUAUUACACCUCUUAAAGA), or *GBA* (CCCAAAAUUUGCUACUUAA). As a nonsilencing control, the Silencer Select Negative Control #1 siRNA was used. At 72 h after transfection, the medium was discarded, and the cells were retransfected with siRNAs. At 48 h after a second transfection, the cells were harvested for experiments. Before collecting data, we checked that different siRNAs complementary to different portions of the gene showed the phenomena that we primarily targeted.

To evaluate cell membrane damage, LDH assay was performed using the Cytotoxicity LDH Assay Kit-WST (Dojindo Molecular Technologies) (10). Cells were treated with the same conditions as the corresponding experiments. As a positive control, lysis buffer containing Triton X-100 was added to cells. LDH release was calculated as a percentage relative to a positive control.

### Chemical treatments

For induction of autophagy, cells were treated with 5 μM rapamycin for 24 h. For inhibition of lysosomal proteases, cells were treated with a lysosomal protease inhibitor cocktail containing E64-days, pepstatin A, and leupeptin at 5 μg/dl each for 24 h. For inhibition of vacuolar-type H (+)-ATPase and autophagosome-lysosome fusion, cells were treated with 10 nM bafilomycin A1 for 24 h. For chemical treatments of siRNA-transfected cells, cells were replaced from media to siRNAs-depleted fresh Opti-MEM after completing a second transfection, and then they were treated with chemical agents by the same condition. In immunofluorescence analysis for morphological changes of lysosomes, cells were treated with 1 μM L-leucyl-L-leucine methyl ester for 1 h as a positive control.

### Preparation of cell lysates and conditioned media

Cells were collected by PBS (10 mM phosphate, 137 mM NaCl, and 2.7 mM KCl) and centrifuged at 6000*g* for 5 min at 4 °C (10). The supernatant was collected in buffer A [20 mM Tris-HCl, pH 7.4, 150 mM NaCl, 1% Triton X-100, 10% glycerol, 1 × protease inhibitor cocktail (Roche Diagnostic), 1 mM EDTA, 5 mM NaF, 1 mM Na3VO4, 1 × phosSTOP (Roche Diagnostic)], sonicated at 30W for 1 s 10 times, and kept on ice for 30 min. After centrifugation at 100,000*g* for 30 min at 4 °C, the supernatant was collected as soluble fractions and stored at −80 °C. After washing pellets with buffer A, resultant pellets were dissolved in the equal aliquot of the solution containing 8 M urea and 2% SDS and sonicated at 30 W for 1 s 10 times. After centrifugation at 100,000*g* for 30 min at 20 °C, the resultant supernatant was collected as insoluble fractions and stored at −80 °C.

To obtain conditioned media (CM), cells were replaced from growth media to Opti-MEM and incubated for 24 h (10). To perform TCA precipitation, collected CM was centrifuged at 6000*g* for 5 min to remove cell debris. Immediately, CM was added with one-fourth volume of 100% TCA, incubated for 30 min on ice, and centrifuged at 20,000*g* for 30 min at 4 °C. The pellet was washed three times with 1 ml of cold acetone, air dried, and dissolved in 100 μl of Laemmli's sample buffer containing 2.5% 2-mercaptoethanol. For fractionation of CM, the TCA-precipitates were dissolved in 100 μl of 1% Triton X-100 containing buffer A, sonicated at 30W for 1 s 10 times, and kept on ice for 30 min. After centrifugation at 100,000*g* for 30 min at 4 °C, the supernatant was collected as soluble fractions. After washing pellets with buffer A, resultant pellets were dissolved in 100 μl of solution containing 8 M urea and 2% SDS and sonicated at 30 W for 1 s 10 times. After centrifugation at 100,000*g* for 30 min at 20 °C, the resultant supernatant was collected as insoluble fractions. Soluble and insoluble fractions of CM were denatured by incubation at 95 °C for 5 min in Laemmli's sample buffer containing 2.5% 2-mercaptoethanol and stored at −80 °C.

### Western blotting

Samples were denatured by incubation at 95 °C for 5 min in Laemmli's sample buffer containing 2.5% 2-mercaptoethanol. Equal amounts of denatured samples were subjected to 13.5% polyacrylamide gel and then transferred to PVDF membranes (0.45 μm pore size, Immobilon-P, Merck Millipore) as described previously (48). To prevent detachment of proteins from membranes, the transferred membrane was incubated in PBS containing 4% paraformaldehyde (48). After incubation, the membrane was washed three times for 10 min in Tris-buffered saline (25 mM Tris-HCl, pH 7.4, 137 mM NaCl, and 2.7 mM KCl) containing 0.05% (v/v) Tween 20 (TBS-T). The membrane was blocked by TBS-T containing 5% skim milk for 30 min, incubated in TBS-T containing 2.5% skim milk and primary antibody overnight at 4 °C, and further incubated in the same buffer containing the corresponding secondary antibody overnight at 4 °C. To visualize the signal, membranes were treated with ECL plus (Thermo Fisher Scientific) for detecting insoluble α-syn of cell lysates and secreted proteins of CM. In other signals, membranes were treated with ECL (Thermo Fisher Scientific). Signals were visualized by a CCD camera, Fusion FX7 (Vilber Lourmat). Signal intensities were analyzed with a Quantity One software (Bio-Rad). The following primary antibodies were used: anti-α-syn (mouse mAb, 1:2500, 42/α-syn, BD Transduction Laboratories, lot: 7243572), anti-β-actin (mouse mAb, 1:10,000, AC-15, Sigma-Aldrich, lot: 0000120485), anti-LC3 (mouse mAb,1:2000, 8E10, MBL, lot: 008), anti-SQSTM/p62 (rabbit mAb, 1:2,000, #5114, Cell Signaling Technology, lot: 6), anti-CTSB (rabbit mAb, 1:2,000, D1C7Y, Cell Signaling Technology, lot: 4), anti-phospho-AMPKα (Thr172) (rabbit mAb, 1:2,000, 40H9, Cell Signaling Technology, lot: 21) (49), anti-phospho-RPS6KB1 (Thr389) (rabbit mAb, 1:2,000, 108D2, Cell Signaling Technology, lot: 12) (50), anti-ATG5 (mouse mAb, 1:2,000, 4D3, MBL, lot: 014), anti-beclin 1 (rabbit mAb, 1:2,000, EPR19662, Abcam, lot: GR3254931-3), anti-STX4 (rabbit mAb, 1:2,000, ARC2113, Thermo Fisher Scientific, lot: XD3557222), anti-SNAP23 (rabbit mAb, 1:2,000, JA73-15, Thermo-Fisher Scientific, lot: XF36147226), anti-RAB8A (rabbit mAb, 1:2,000, EPR14873, Abcam, lot: GR3287452-14), anti-FIP200 (rabbit mAb, 1:2,000, D10D11, Cell Signaling Technology, lot: 2), anti-GRASP55 (rabbit mAb, 1:2,000, F8L6I, Cell Signaling Technology, lot: 1), anti-GBA (rabbit mAb, 1:2,000, E2R1L, Cell Signaling Technology, lot: 1) antibodies. As secondary antibodies, we used peroxidase-conjugated donkey anti-mouse IgG (H + L) (1:5,000, Jackson ImmunoResearch Inc) and peroxidase-conjugated goat anti-rabbit IgG (H + L) (1:5,000, Jackson ImmunoResearch Inc).

### Immunocytochemistry

Cells were plated on Geltrex (Thermo Fisher Scientific)-coated square glass coverslips at a density of 1.0 × 10^5^ cells per well in 6-well plates. After transfection with siRNAs or treatments with chemical agents, cells were fixed by methanol for 5 min at −20 °C. After blocked with TBS-T containing 5% skim milk for 30 min at room temperature, coverslips were incubated in TBS-T containing 2.5% skim milk and a mixture of primary antibodies overnight at 4 °C. After washing, coverslips were incubated in TBS-T containing 2.5% skim milk and a mixture of secondary antibodies for 4 h at 37 °C. Images were acquired on laser-scanning confocal microscope (TCS SP8, Leica Microsystems) (10). For analyzing lysosome size, we randomly selected five cells in anti-LAMP1 antibody-stained samples, captured images, and automatically measured area of lysosomes on the images by using the free ImageJ software. The following primary antibodies were used: anti-α-syn (chicken polyclonal, 1:500, C-1795-50, Biosensis, lot: C-1795-300-201807-MC), anti-LC3 (rabbit polyclonal, 1:100, PM036, MBL, lot: 037), anti-LC3 (mouse mAb, 1:100, 8E10, MBL, lot: 008), anti-RAB5A (mouse mAb, 1:100, E6N8S, Cell Signaling Technology, lot: 3), anti-RAB7 (mouse mAb, 1:100, E907E, Cell Signaling Technology, lot: 2), anti-LAMP1 (mouse mAb, 1:100, D401S, Cell Signaling Technology, lot: 4), anti-LAMP1 (rabbit mAb, 1:100, D2D11, Cell Signaling Technology, lot: 8), and anti-galectin 3 (rat mAb, 1:100, M3/38, Santa Cruz Biotechnology, lot: F0524) antibodies. The following secondary antibodies were used: Alexa Fluor 405 goat anti-chicken IgY (H + L) (1:500, Abcam), Alexa Fluor 488 donkey anti-mouse IgG (H + L) (1:500, Jackson ImmunoResearch Inc), Alexa Fluor 594 donkey anti-rabbit IgG (H + L) (1:500, Jackson ImmunoResearch Inc), and Alexa Fluor 594 goat anti-rat IgG (H + L) (1:500, Abcam).

### Transmission electron microscopy

Collected cells were fixed with 0.05 M cacodylate buffer (pH 7.2) containing 2% glutaraldehyde at 4 °C for 3 h (51). Fixed cells were washed five times with 0.05 M cacodylate buffer and secondarily fixed with 0.06 M cacodylate buffer containing 1% osmium tetroxide for 2 h. The cells were dehydrated with ethanol and embedded in epoxy resin. Ultra-thin sections (100-nm-thick) were obtained using an ultramicrotome (ULTRACUT-N, Nissei Sangyo), and they were mounted on a nickel grid supported by a carbon-coated collodion film. The sections were stained with 4% (w/v) uranyl acetate for 10 min followed by lead citrate for 3 min. All sections were observed with a Hitachi HT7800 transmission electron microscopy.

### Statistical analysis

Statistical analysis was performed using the SPSS software (version 17, IBM). Comparison of two groups was performed by unpaired *t* test. Multiple comparisons above three groups were performed by one-way ANOVA with Bonferroni *post hoc* test, when the variances were homogenous. In the case of nonhomogeneity of variances, comparisons were performed by Welch-ANOVA with Games–Howell *post hoc* test. Data are expressed as mean ± SD, and *p* < 0.05 was considered statistically significant.

## Data availability

All unique reagents and materials reported in this study will be made available upon reasonable request without restrictions. All data are contained within the article.

## Supporting information

This article contains supporting information.

## Conflict of interest

The authors declare that they have no conflicts of interest with the contents of this article.
