# Supplementary material for: Secretory autophagy mediates lysosomal and autophagic degradation for α-synuclein proteostasis
Source: J Biol Chem. 2025 Jul 17;301(8):110474. doi: 10.1016/j.jbc.2025.110474 (PMC12362094; doi:10.1016/j.jbc.2025.110474)
Supplement: Supplementary Material [file mmc1.docx]

**Supporting information**

**Secretory autophagy mediates lysosomal and autophagic degradation for α-synuclein proteostasis**

Taiki Sawai^1^, Yoshitsugu Nakamura^1^, and Shigeki Arawaka^1*^

^1^Department of Internal Medicine IV, Division of Neurology, Osaka Medical and Pharmaceutical University Faculty of Medicine. 2-7 Daigaku-machi, Takatsuki, Osaka 569-8686, Japan

^⁎^Address correspondence to Shigeki Arawaka, Department of Internal Medicine IV, Division of Neurology, Osaka Medical and Pharmaceutical University Faculty of Medicine. 2-7 Daigaku-machi, Takatsuki, Osaka 569-8686, Japan

Tel: +81-72-683-1221; FAX: +81-72-684-7087; E-mail: [shigeki.arawaka@ompu.ac.jp](mailto:shigeki.arawaka@ompu.ac.jp).

**Contents**:

1. Figure S1: The expression levels of target genes by siRNA-mediated knockdowns.

2. Figure S2: Analysis of colocalization between cathepsin B and RAB5, RAB7 or LAMP1-positive structures in *ATG5* knockdown wt-αS/SH cells.

3. Figure S3: Analysis of colocalization between LC3 and LAMP1-positive structures in wt-αS/SH cells.

4. Figure S4: Effects of *SNAP23* knockdown on secretion of α-synuclein with altered solubility in wt-αS/SH cells.

5. Figure S5: Effects of bafilomycin A1 on the *SNAP23* knockdown-induced changes of autophagic flux and lysosomal function.

6. Figure S6: Immunofluorescence confocal microscopy analysis of galectin 3 and LAMP1-positive structures in *ATG5* knockdown wt-αS/SH cells.

7. Figure S7: Immunofluorescence confocal microscopy analysis of galectin 3, LC3, and LAMP1-positive structures in *SNAP23* knockdown wt-αS/SH cells.

8. Figure S8: Effects of *GRASP55* knockdown on autophagic secretion, autophagic flux for degradation, and lysosome function.

**
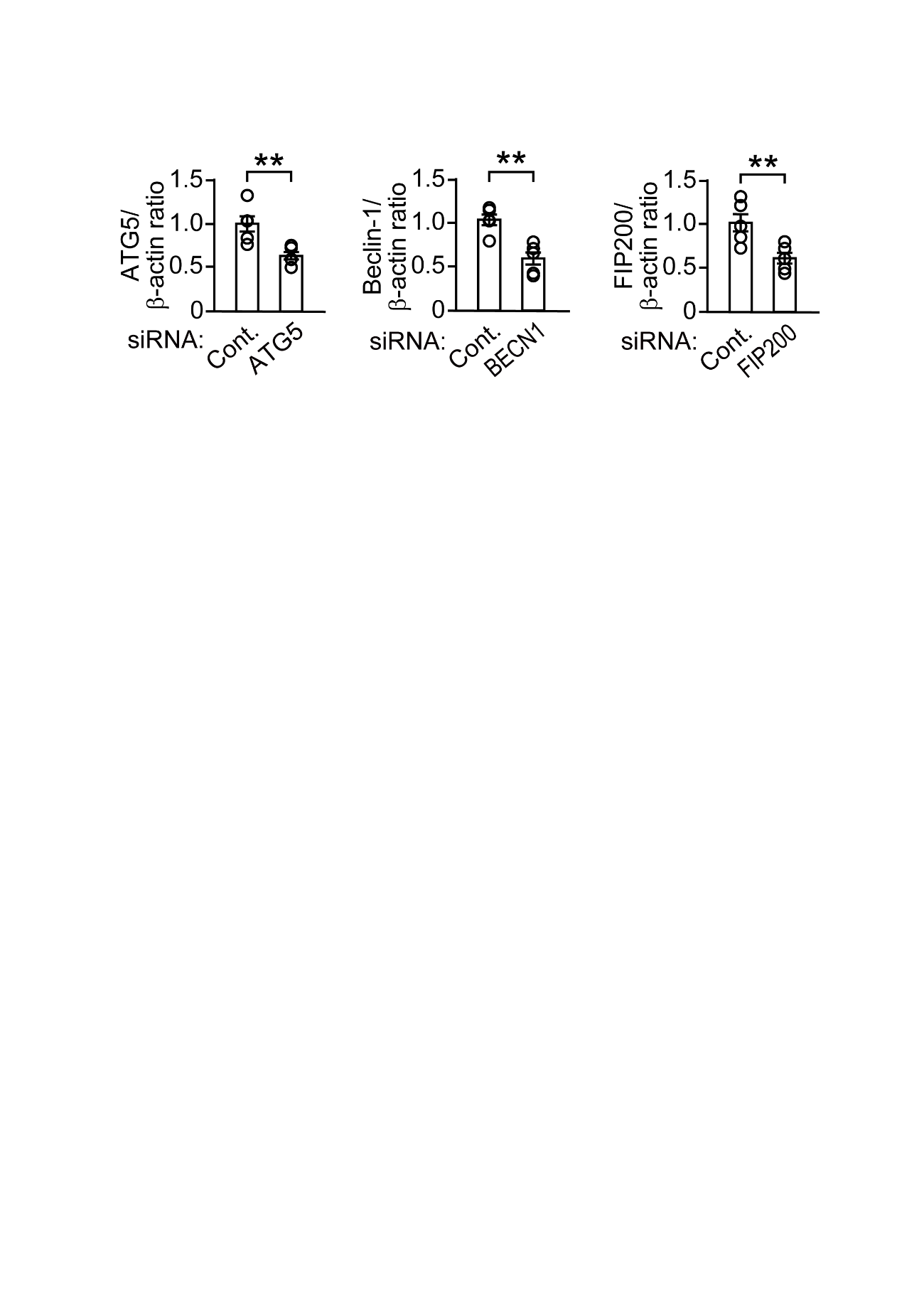
Supplementary figure 1**

**Figure S1. The expression levels of target genes by siRNA-mediated knockdowns in SH-SY5Y cells stably expressing wild-type α-synuclein (wt-αS/SH).**

Graphs show ratios of the expression levels of target genes to those of β-actin, compared with control siRNA. These data are based on the blots of Fig. 1. Left, middle, and right graphs show the data of *ATG5*, *BECN1*, and *FIP200* knockdowns, respectively. Data represent mean ± SD. Data are analyzed by unpaired *t* test. **p < 0.01. Cont., control.

**
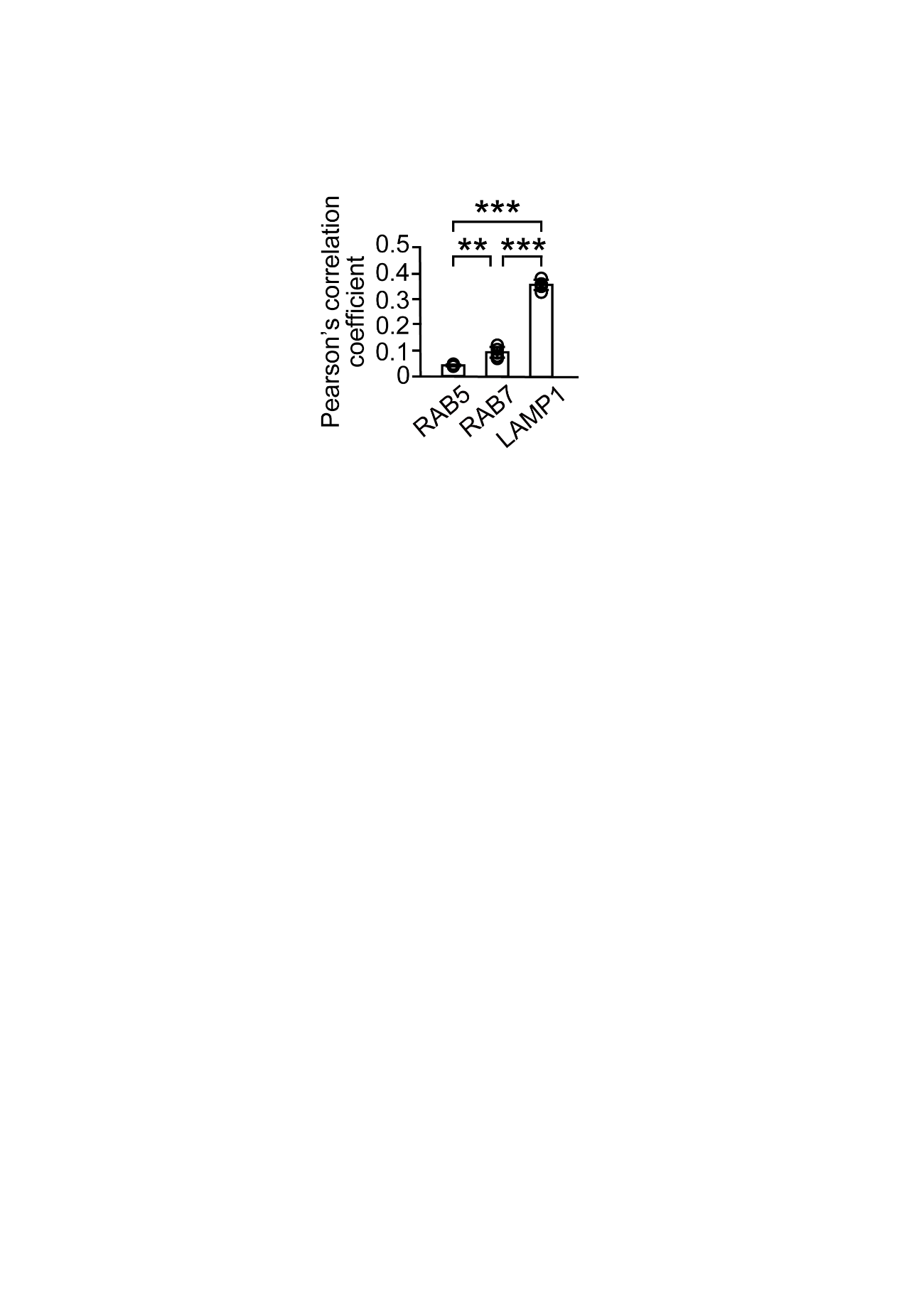
Supplementary figure 2**

**Figure S2. Analysis of colocalization between cathepsin B and RAB5, RAB7 or LAMP1-positive structures in *ATG5* knockdown wt-αS/SH cells.**

Pearson’s correlation coefficients were calculated by the software attached to the laser-scanning confocal microscope (TCS SP8, Leica Microsystems). The values were obtained by randomly mesureing five images per each double staining in Fig. 2B. Data represent mean ± SD and were analyzed by one-way ANOVA with Bonferroni *post hoc* tests. **p < 0.01, ***p < 0.001.

**
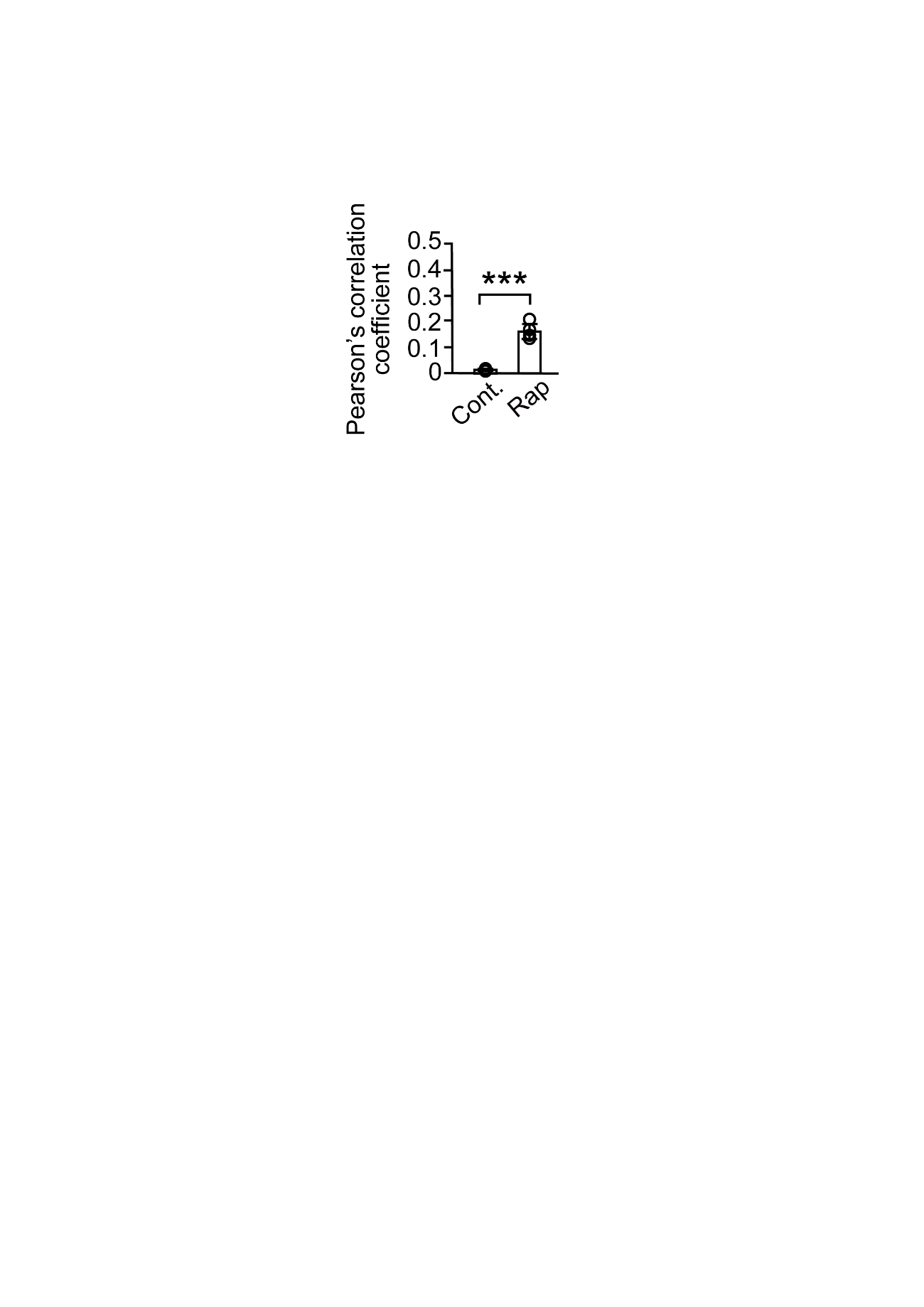
Supplementary figure 3**

**Figure S3. Analysis of colocalization between LC3 and LAMP1-positive structures in wt-αS/SH cells.**

Pearson’s correlation coefficients were calculated by the software attached to the laser-scanning confocal microscope (TCS SP8, Leica Microsystems). The values were obtained by randomly mesureing five images per each double staining in Fig. 3A. Data represent mean ± SD and were analyzed by unpaired *t* test. ***p < 0.001. Cont., control; Rap, rapamycin.

**Supplementary figure 4**

**
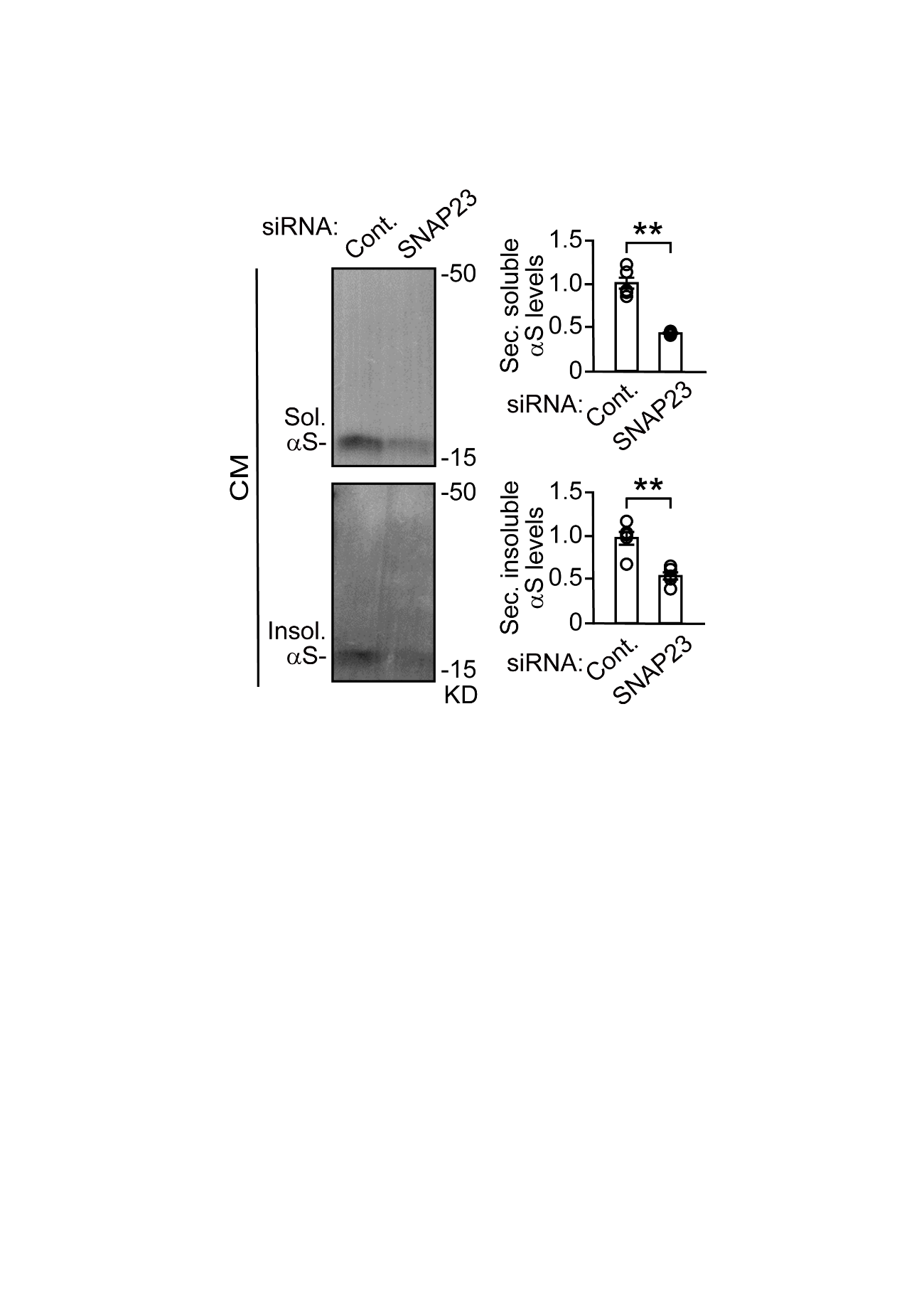
**

**Figure S4. Effects of *SNAP23* knockdown on secretion of α-synuclein with altered solubility in wt-αS/SH cells.**

Conditioned media from control or *SNAP23* siRNA knockdown cells were separated into 1% Triton X-100-soluble and 1% Triton X-100-insoluble fractions. The fractionation protocol is seen in the Experimental procedures. These fractions were blotted to detect indicated proteins (n = 5). Right graphs show quantitative comparisons of the relative levels and ratios of target proteins. Data represent mean ± SD. Data are analyzed by unpaired *t* test. **p < 0.01. CM; conditioned media, Cont.; control, Insol. αS; insoluble α-synuclein, Sol. αS; soluble α-synuclein.

**Supplementary figure 5**

**
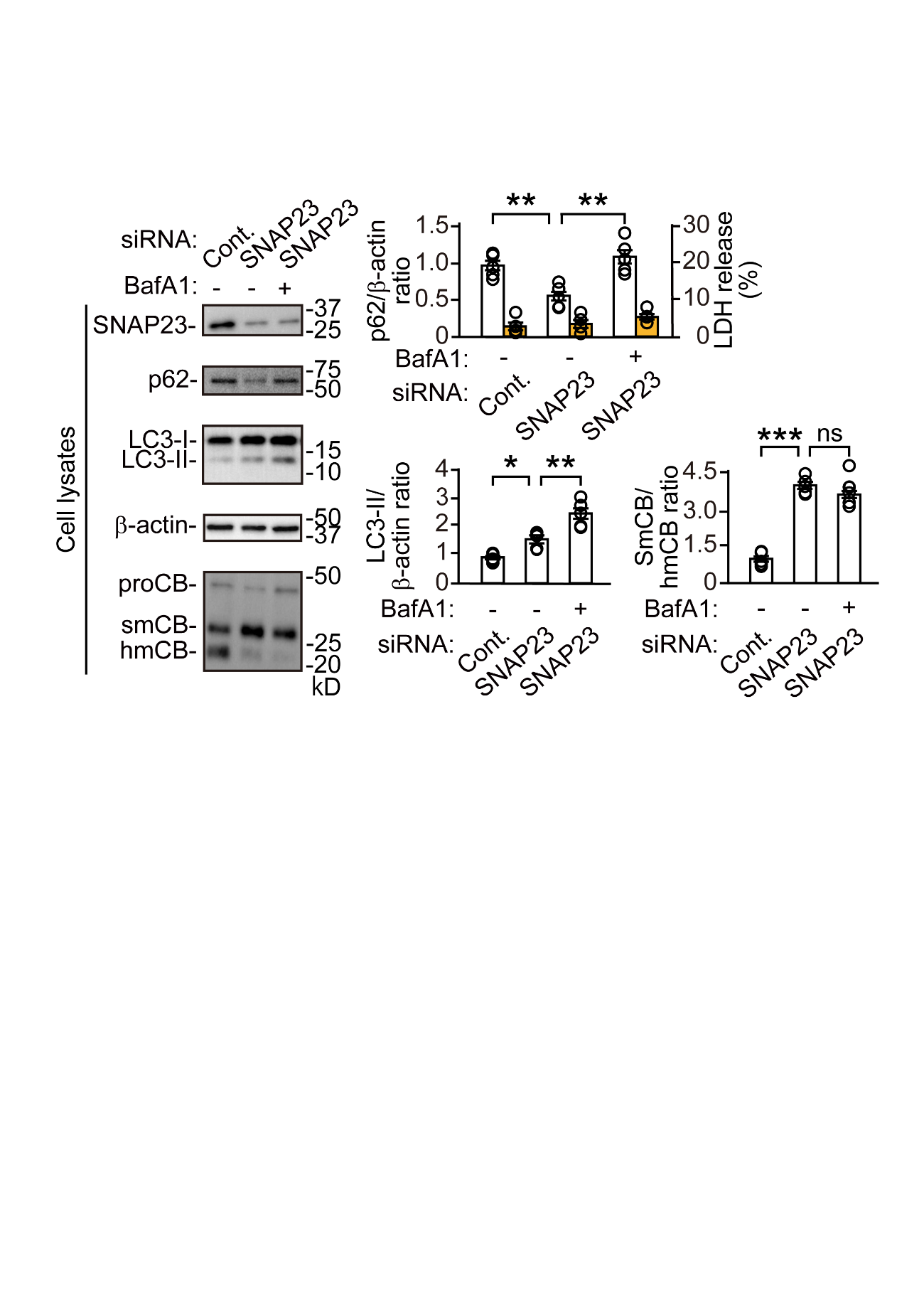
**

**Figure S5. Effects of bafilomycin A1 on the *SNAP23* knockdown-induced changes of autophagic flux and lysosomal function in wt-αS/SH cells.**

Cells were transfected with siRNA oligonucleotides against non-silencing control or *SNAP23*. The cells were treated with 10 nM bafilomycin A1 or the equivalent volume of vehicle as a control for 24 h. Conditioned media and cell lysates from control or *SNAP23* knockdown cells were blotted to detect indicated proteins (n = 5). Lactate dehydrogenase (LDH) release was measured on each condition (n = 4). Right graphs show quantitative comparisons of the relative levels and ratios of target proteins. Percentages of LDH release to positive controls are shown as orange columns*.* Data represent mean ± SD and were analyzed by one-way ANOVA with Bonferroni post hoc tests. *p < 0.05, **p < 0.01, ***p < 0.001. BafA1, bafilomycin A1; Cont., control; hmCB, heavy-chain mature cathepsin B; ns, not significant; proCB, pro-cathepsin B; smCB, single-chain mature cathepsin B.

**
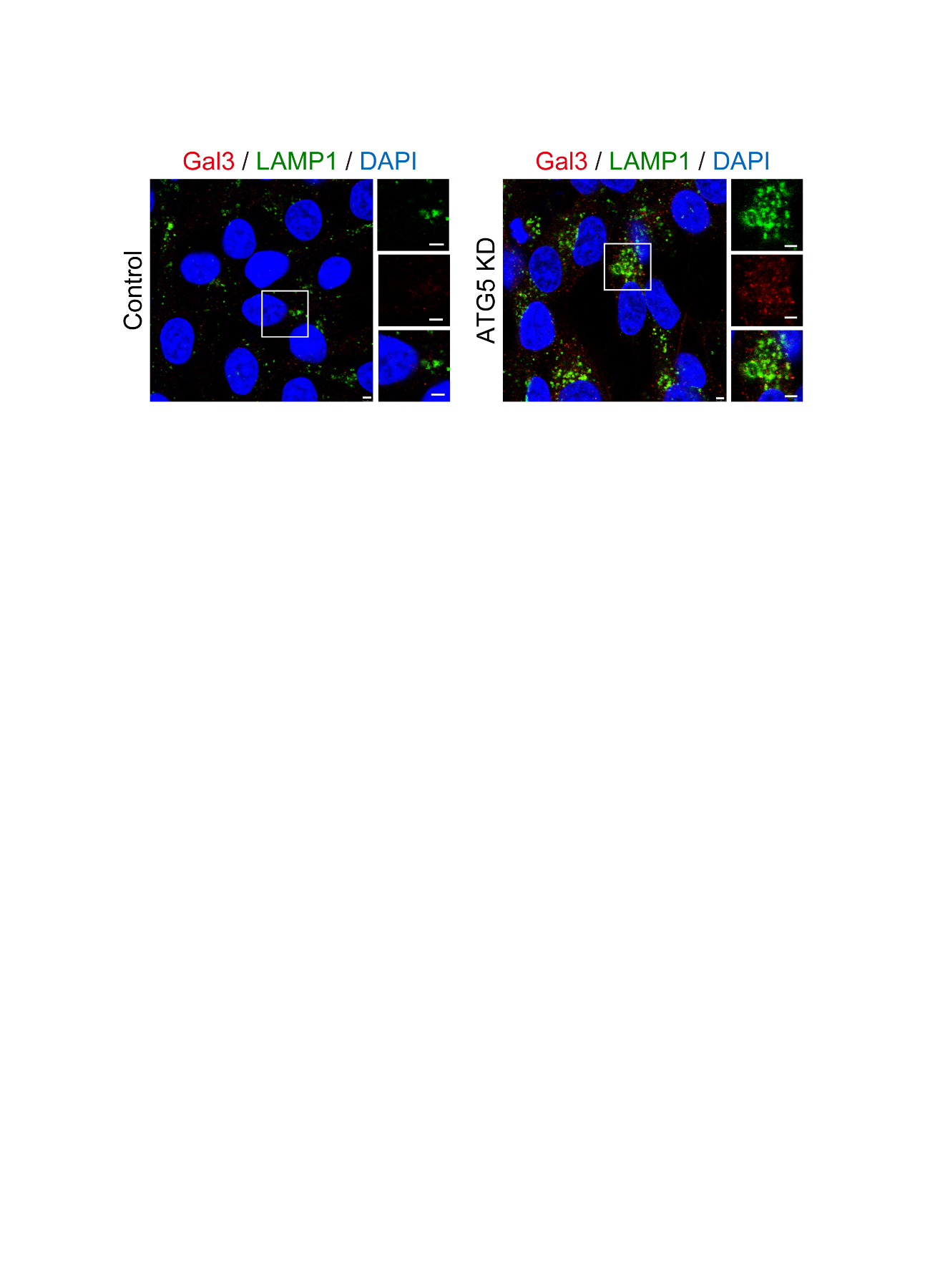
Supplementary figure 6**

**Figure S6. Immunofluorescence confocal microscopy analysis of galectin 3 and LAMP1-positive structures in *ATG5* knockdown wt-αS/SH cells.**

Control or *ATG5* knockdown cells were stained with anti-LAMP1 (green, right upper panels), anti-galectin 3 (red, right middle panels), and DAPI (blue). Left micrographs show merged images. Right panels of micrographs show images of white squared areas. Scale bar: 3 μm. Gal3, galectin 3; KD, knockdown.

**
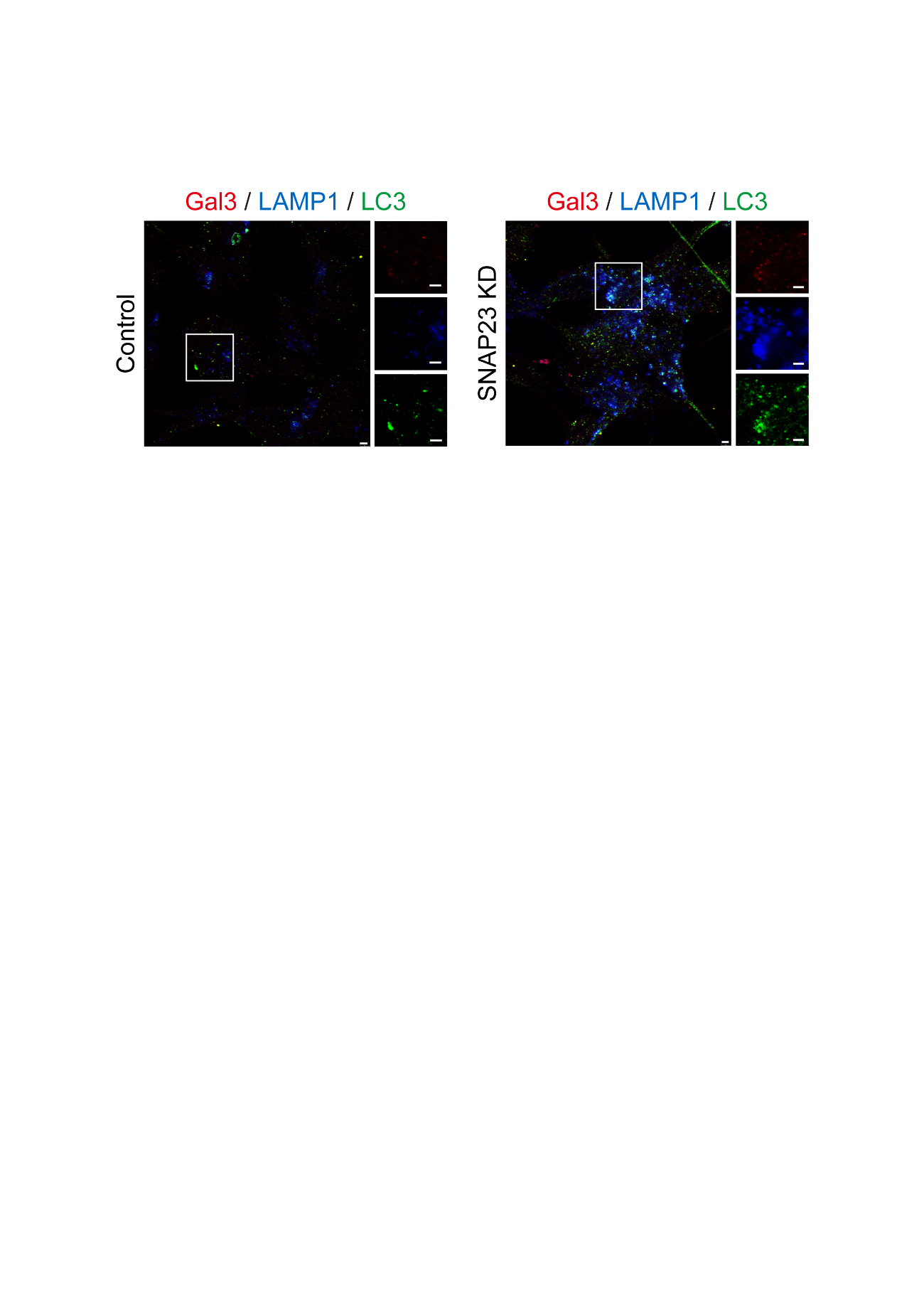
Supplementary figure 7**

**Figure S7. Immunofluorescence confocal microscopy analysis of galectin 3, LC3, and LAMP1-positive structures in *SNAP23* knockdown wt-αS/SH cells.**

Control or *SNAP23* knockdown cells were stained with anti-galectin 3 (red, right upper panels), anti-LAMP1 (blue, right middle panels) and anti-LC3 (green, right lower panels). Left micrographs show triple merged images. Right panels of micrographs show images of white squared areas. Scale bar: 3 μm. Gal3, galectin 3; KD, knockdown.

**
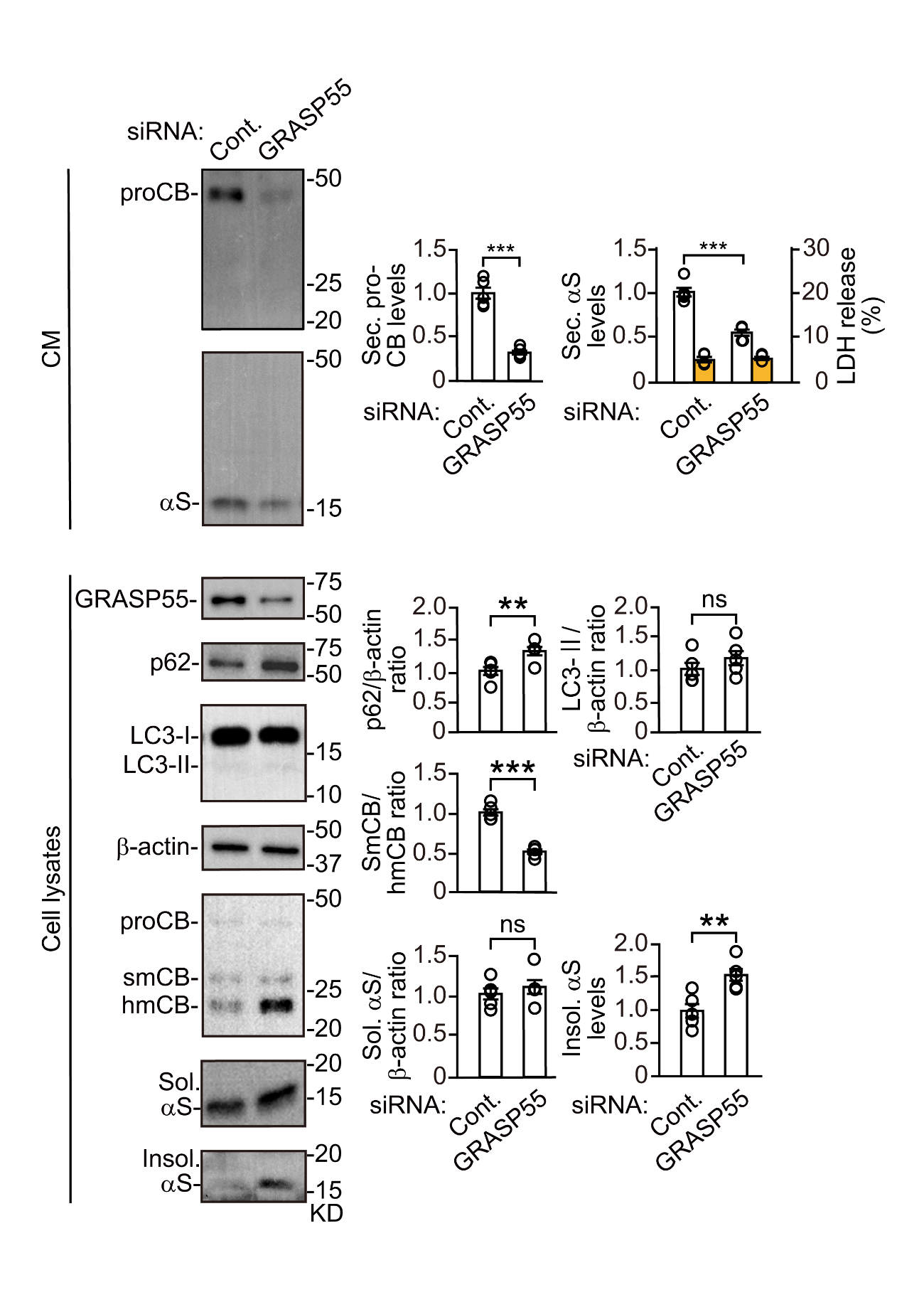
Supplementary figure 8**

**Figure S8. Effects of *GRASP55* knockdown on autophagic secretion, autophagic flux for degradation, and lysosome function in wt-αS/SH cells.**

Cells were transfected with siRNA oligonucleotides against non-silencing control or *GRASP55*. Conditioned media and cell lysates from control or *GRASP55* knockdown cells were blotted to detect indicated proteins (n = 5). LDH release was measured on each condition (n = 4). Right graphs show quantitative comparisons of the relative levels and ratios of target proteins. Percentages of LDH release to positive controls are shown as orange columns. Data represent mean ± SD. Data were analyzed by unpaired *t* test. **p < 0.01, ***p < 0.001. αS, α-synuclein; CM, conditioned media; Cont., control; hmCB, heavy chain mature cathepsin B; Insol. αS, insoluble α-synuclein; ns, not significant; proCB, pro-cathepsin B; smCB, single chain mature cathepsin B; Sol. αS, soluble α-synuclein.
